# Allosteric regulators selectively prevent Ca^2+^-feedback of Ca_V_ and Na_V_ channels

**DOI:** 10.7554/eLife.35222

**Published:** 2018-09-10

**Authors:** Jacqueline Niu, Ivy E Dick, Wanjun Yang, Moradeke A Bamgboye, David T Yue, Gordon Tomaselli, Takanari Inoue, Manu Ben-Johny

**Affiliations:** 1 Department of Biomedical EngineeringJohns Hopkins UniversityBaltimoreUnited States; 2 Department of PhysiologyUniversity of MarylandBaltimoreUnited States; 3 Department of CardiologyJohns Hopkins UniversityBaltimoreUnited States; 4 Department of Cell BiologyJohns Hopkins UniversityBaltimoreUnited States; 5 Center for Cell Dynamics, Institute for Basic Biomedical SciencesJohns Hopkins UniversityBaltimoreUnited States; 6 Department of Physiology and Cellular Biophysics, College of Physicians and SurgeonsColumbia UniversityNew YorkUnited States; Northwestern UniversityUnited States; The University of Texas at AustinUnited States

**Keywords:** Ca channels, Na channels, calmodulin, fibroblast growth factor homologous factors, SH3 and cysteine rich domain proteins, calcium regulation, Other

## Abstract

Calmodulin (CaM) serves as a pervasive regulatory subunit of Ca_V_1, Ca_V_2, and Na_V_1 channels, exploiting a functionally conserved carboxy-tail element to afford dynamic Ca^2+^-feedback of cellular excitability in neurons and cardiomyocytes. Yet this modularity counters functional adaptability, as global changes in ambient CaM indiscriminately alter its targets. Here, we demonstrate that two structurally unrelated proteins, SH3 and cysteine-rich domain (stac) and fibroblast growth factor homologous factors (fhf) selectively diminish Ca^2+^/CaM-regulation of Ca_V_1 and Na_V_1 families, respectively. The two proteins operate on allosteric sites within upstream portions of respective channel carboxy-tails, distinct from the CaM-binding interface. Generalizing this mechanism, insertion of a short RxxK binding motif into Ca_V_1.3 carboxy-tail confers synthetic switching of CaM regulation by Mona SH3 domain. Overall, our findings identify a general class of auxiliary proteins that modify Ca^2+^/CaM signaling to individual targets allowing spatial and temporal orchestration of feedback, and outline strategies for engineering Ca^2+^/CaM signaling to individual targets.

## Introduction

Supporting vital biological functions, voltage-gated calcium (Ca_V_1 and Ca_V_2) and sodium (Na_V_1) channels are tuned by the Ca^2+^-binding protein, calmodulin (CaM) (Ben-Johny et al., 2015; Catterall et al., 2017; Saimi and Kung, 2002). Na_V_1 supports action potential initiation and propagation (Hille, 2001), while Ca_V_1/2 initiate muscle contraction, neurotransmission, and gene transcription (Berridge et al., 2000; Clapham, 2007; Maier and Bers, 2002). Despite divergent functions, these channel families share a conserved carboxy-tail element, termed Ca^2+^-inactivating (CI) module, that harbors CaM. Functionally, the CI module confers dynamic Ca^2+^-dependent regulation to Ca_V_1, Ca_V_2, and Na_V_1 that manifests as either inactivation (CDI) or facilitation (CDF), negative and positive feedback, respectively (Ben-Johny et al., 2015; Minor and Findeisen, 2010). Yet, this modularity poses a challenge – mechanisms that tune Ca^2+^/CaM-feedback must distinguish between structurally similar targets. Global inhibition of CaM indiscriminately alters many processes (Persechini and Stemmer, 2002). Given the abundance of CaM-regulated proteins, mechanisms that adjust CaM signaling to individual targets are crucial (Marshall et al., 2015; Saimi and Kung, 2002). Physiologically, Ca^2+^-regulation of Ca_V_1 is critical for cardiac electrical stability (Limpitikul et al., 2014; Mahajan et al., 2008), rhythmicity of oscillatory neurons (Chan et al., 2007; Huang et al., 2012), and vesicle release at ribbon synapses (Joiner and Lee, 2015). Ca_V_2 modulation contributes to short-term synaptic plasticity and spatial learning (Adams et al., 2010; Jackman and Regehr, 2017; Nanou et al., 2016), while Na_V_1 modulation tunes excitability of skeletal and cardiac muscle (Pitt and Lee, 2016; Van Petegem et al., 2012). Consequently, aberrant channel regulation underlies numerous maladies including cardiac arrhythmias (Venetucci et al., 2012; Zimmer and Surber, 2008), neurological and neuropsychiatric disorders (Adams and Snutch, 2007; Striessnig et al., 2010; Zamponi, 2016), and skeletal myotonia (Cannon, 2015).

Src homology 3 (SH3) and cysteine-rich domain (C1) proteins (stac) have emerged as attractive candidates that modulate Ca_V_ gating and trafficking (Polster et al., 2015; Suzuki et al., 1996). Initially identified in association with congenital skeletal myopathies as a structural protein that abets Ca_V_1.1 plasmalemmal trafficking (Horstick et al., 2013; Linsley et al., 2017c; Polster et al., 2015), stac also suppresses Ca_V_1.2 CDI (Campiglio et al., 2018; Wong King Yuen et al., 2017). Even so, the specificity of stac in tuning Ca^2+^-regulation of the broader Ca_V_/Na_V_ family, the underlying elementary mechanisms, and molecular determinants remain to be fully elucidated (Wong King Yuen et al., 2017). Stac isoforms (stac1-3) share a common architecture containing a C1 and two SH3 domains fused via a linker, and exhibit tissue-specific expression (Suzuki et al., 1996). Stac1/2 are expressed throughout the brain (Nelson et al., 2013; Suzuki et al., 1996), the peripheral nervous system (Legha et al., 2010), the retina (Wilhelm et al., 2014), and the inner ear (Cai et al., 2015), while stac3 is limited to the skeletal muscle (Nelson et al., 2013). Resolving mechanisms by which stac modulates Ca_V_ may furnish long-sought physiological insights (Suzuki et al., 1996).

Evolutionarily distinct from stac, fibroblast growth factor (fgf) homologous factors (fhf1-4, fgf11-14) are unconventional fgf that lack a secretory signal and serve as intracellular regulators of Na_V_ gating and trafficking (Goldfarb, 2005; Pablo and Pitt, 2016). Curiously, fhf interacts with the Na_V_ CI module in close proximity to the CaM binding interface, suggesting interplay between these modulators (Wang et al., 2012; Wang et al., 2011b). Yet, functionally, fhf is thought to modulate only voltage-dependent fast inactivation (Goldfarb et al., 2007; Lou et al., 2005; Wang et al., 2011a), with changes in Ca^2+^-regulation unknown. Fhf isoforms are differentially expressed in the brain (Smallwood et al., 1996; Yan et al., 2014), peripheral nervous system (Ornitz and Itoh, 2001), and cardiac (Wei et al., 2011) and skeletal muscle (Kraner et al., 2012). Genetic variation in fhf4 is linked to spinocerebellar ataxia 27 (Coebergh et al., 2014) and fhf1 to cardiac arrhythmias (Wei et al., 2011), hinting at their relevance for regulating neuronal and cardiac excitability.

By leveraging synergistic insights from Ca_V_ and Na_V_ channels, we demonstrate that stac selectively diminishes Ca^2+^-regulation of Ca_V_1. In-depth analysis shows that stac binds to a distinct channel interface from CaM and uses an allosteric mechanism to lock Ca_V_1 into a high open probability (*P*_O_) gating mode. We further localize a minimal motif that recapitulates stac modulation of Ca_V_1 gating. Paralleling stac-effect on Ca_V_1, fhf reduces CDI of Na_V_1 with no effect on Ca_V_1. In all, our findings point to a general class of auxiliary proteins that intercept CaM signaling to individual targets, allowing spatial and temporal orchestration of Ca^2+^-feedback.

## Results

### Stac selectively suppresses Ca^2+^-feedback of Ca_V_1 channels

**Figure 1.**
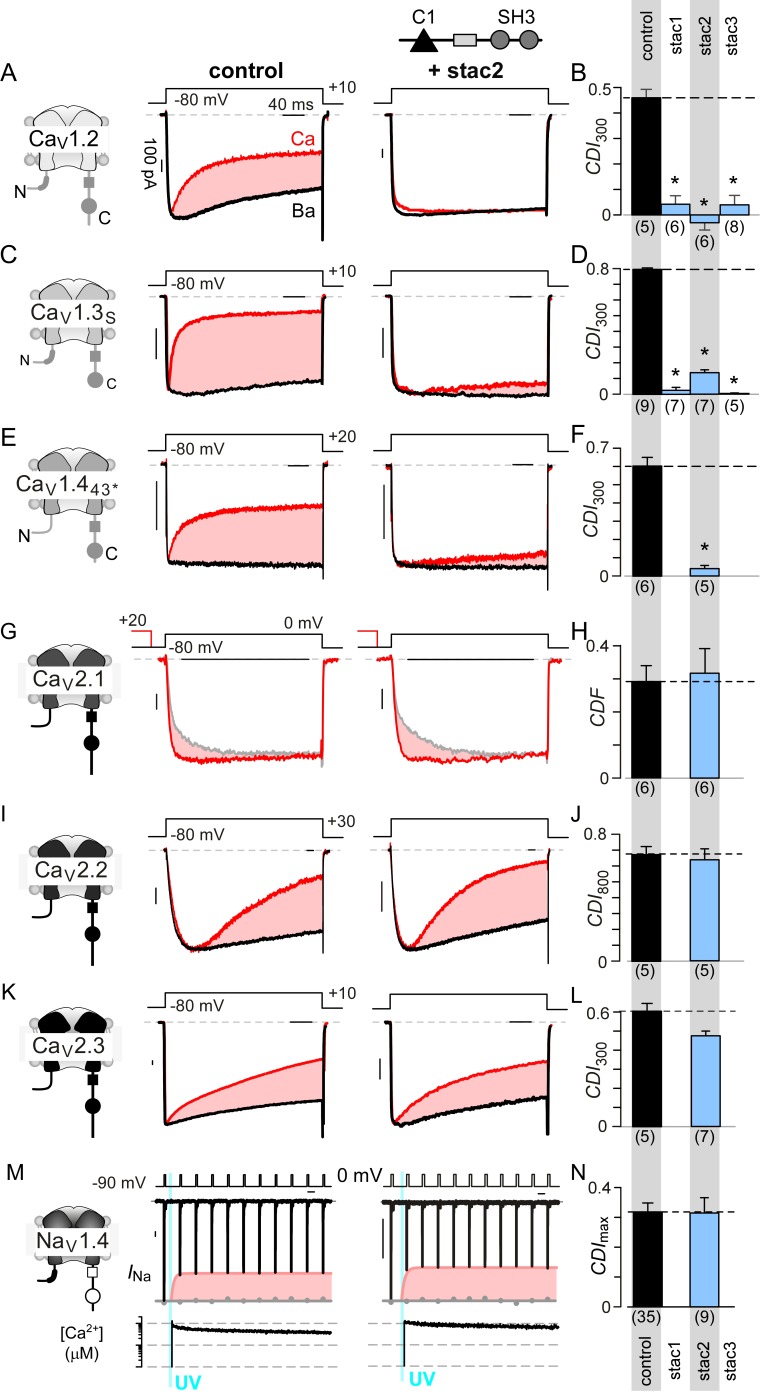
Stac specifically abolishes Ca^2+^/CaM-regulation in Ca_V_1 channels. (**A**) Stac2 diminishes CDI of Ca_V_1.2. Left, cartoon schematic shows Ca_V_1.2. Middle, exemplar current traces evoked in response to +10 mV voltage-step shows robust CDI (rose shaded area) evident as enhanced current decay with Ca^2+^ (red) versus Ba^2+^ (black) as the charge carrier. Right, stac2 abolishes CDI. Steady-state levels of inactivation are assessed as the fraction of peak current remaining following 300 ms depolarization (*r*_Ca_ and *r*_Ba_) and CDI = 1 – *r*_Ca_/*r*_Ba_. (**B**) Bar graph displays population data of CDI_300_ for Ca_V_1.2 in the absence and in the presence of stac1, stac2, or stac3. Dashed line shows baseline CDI in the absence of stac for comparison. Each bar, mean ±S.E.M. obtained from specified number of cells (n). (**C–D**) Stac isoforms suppress CDI of Ca_V_1.3_S_, the canonical short variant, as confirmed by both exemplar traces (**C**) and population data (**D**). Format as in (**A**) and (**B**). (**E–F**) Stac2 abolishes CDI of Ca_V_1.4_43*_ assessed in response to +20 mV test pulse. Format as in (**A**) and (**B**). (**G–H**) Stac2 spares CDF of Ca_V_2.1, as evaluated using a prepulse protocol. An isolated test pulse to 0 mV elicits Ca^2+^ currents with biphasic activation (gray, **G**). With a + 20 mV prepulse, channels are partially facilitated and the slow component of activation is reduced (red, **G**). The area between the two current traces (*ΔQ*), divided by *τ*_slow_, yields facilitation (g). Bar graph plots, CDF = *g*_Ca_ – *g*_Ba_**H**). Each bar, mean ±S.E.M from specified number of cells (n). (**I–J**) Stac2 spares CDI of Ca_V_2.2 assessed in response to +30 mV test pulse. Here, CDI is evaluated following 800 ms of depolarization to accommodate slow inactivation kinetics, yielding CDI_800_. Format as in (**A**) and (**B**). (**K–L**) Stac2 spares CDI of Ca_V_2.3. Format as in (**A**) and (**B**). (**M–N**) Stac2 spares CDI of Na_V_1.4. Both in the absence and presence of stac, Na_V_1.4 peak currents decline following a Ca^2+^ step (rose fit) (**M**). Gray dots, peak currents before uncaging. CDI = 1 – average peak *I*_Na_ of last three to four responses after Ca^2+^ uncaging / peak *I*_Na_ before uncaging. Bar graph plots maximal CDI observed with Ca^2+^ steps > 5 μM (**N**). Each bar, mean ±S.E.M.

We sought to determine stac effect on Ca_V_1, Ca_V_2, and Na_V_1 channels in heterologous systems. Figure 1A shows baseline effects of stac on Ca_V_1.2 (Campiglio et al., 2018; Polster et al., 2015; Wong King Yuen et al., 2017). Devoid of stac, Ca_V_1.2 exhibits CaM-mediated CDI manifesting as enhanced decay of Ca^2+^ (red) versus Ba^2+^ current (black) when elicited using a step depolarization (Figure 1A, middle subpanel). As Ba^2+^ binds CaM poorly (Linse and Forsén, 1995), Ba^2+^-currents furnish a baseline measure of voltage-dependent inactivation (VDI) without CDI. Upon stac2 co-expression, CDI is diminished (Figure 1A, right subpanel). To quantify steady-state extent of inactivation, we measured the fraction of peak Ca^2+^ and Ba^2+^ current remaining after 300 ms depolarization, *r*_Ca_ and *r*_Ba_ (Figure 1—figure supplement 1A). The strength of CDI is quantified as *CDI*_300_ = 1 – *r*_Ca_/*r*_Ba_, the fractional Ca^2+^-dependent component of inactivation. Thus quantified, the population data confirm a reduction in CDI of Ca_V_1.2 with stac2 (p=3.6 × 10^−5^; Figure 1B). Further analysis shows that both stac1 and stac3 isoforms also diminish CDI (p=2.0 × 10^−5^ and 7.1 × 10^−5^, respectively, Figure 1B and Figure 1—figure supplement 1A). Similarly, Ca_V_1.3 short variant (Ca_V_1.3_S_), a close homolog of Ca_V_1.2, exhibits strong baseline CDI that is reduced on co-expression of stac1, stac2, and stac3 (p<1 × 10^−5^; Figure 1C–D and Figure 1—figure supplement 1B). Generalizing this phenomenon, stac2 also reduces CDI of Ca_V_1.4_43*_ (p=3.2 × 10^−5^; Figure 1E–F; Figure 1—figure supplement 1C) (Tan et al., 2012).

**Figure 1—figure supplement 1.**
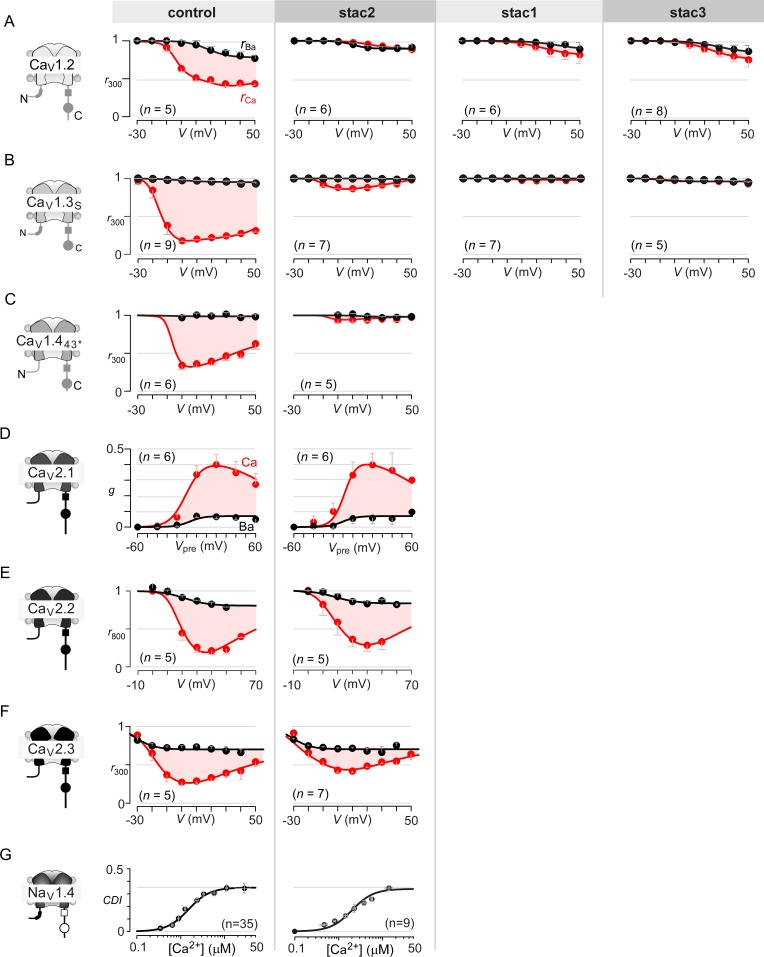
Extended data highlight selectivity of stac in modulating Ca_V_1 versus Ca_V_2 and Na_V_1 channels. (**A**) Stac1-3 isoforms strongly diminish CDI of Ca_V_1.2 (schematized in the far left subpanel). In the absence of stac, Ca_V_1.2 exhibits robust CDI (red shaded area, middle left subpanel). Co-expression of stac2 (middle), stac1 (middle right), and stac3 (far right), all suppress CDI. The steady-state extent of inactivation in Ca^2+^ (red) and Ba^2+^ (black), here, is estimated by the metric *r*_300_, the ratio of current remaining at 300 ms to peak current during a depolarizing pulse, plotted as a function of voltage. Each symbol, mean ±S.E.M. from specified number of cells (n). (**B**) Stac1-3 isoforms suppress CDI of Ca_V_1.3. Format as in (**A**). (**C**) Stac2 co-expression abolishes CDI of Ca_V_1.4_43*_ alternatively spliced variant. Format as in (**A**). (**D**) Stac2 does not alter CDF of Ca_V_2.1. A prepulse protocol is used to estimate CDF. A step depolarization to 0 mV evokes an inward current containing rapid and slow components caused by a superposition of voltage activation and CDF. With a preceding voltage step to 20 mV, sufficient Ca^2+^ enters to partially facilitate the channels, such that the subsequent step to 0 mV has a markedly blunted slow component (Figure 1G). The area between the two current traces (ΔQ), divided by *τ*_slow_, yields the parameter *g*, an estimate of facilitation triggered by the prepulse. Here, *g* plotted as a function of prepulse potentials reveals a U-shaped dependence of Ca^2+^-dependent facilitation. Facilitation is similar in the absence (middle subpanel) and presence of stac2 (right subpanel). (**E**) Stac2 does not suppress CDI of Ca_V_2.2. The steady-state extent of inactivation in Ca^2+^ (red) and Ba^2+^ (black), here, is estimated by the metric *r*_800_, the ratio of current remaining at 800 ms to peak current during a depolarizing pulse, plotted as a function of voltage. Robust CDI is detected under both control conditions (middle subpanel) and stac2 co-expression (right subpanel). (**F**) Similarly, CDI of Ca_V_2.3 is not greatly affected. Format as in (**A**). (**G**) Stac2 spares CDI of Na_V_1.4. Mean data for CDI are plotted versus Ca^2+^-step amplitude. CDI = 1 − average peak *I*_Na_ of last three to four responses after Ca^2+^uncaging/peak *I*_Na_ before uncaging. Symbols, mean ±S.E.M. of ∼four uncaging events compiled from denoted number of cells (n).

Encouraged by its pervasiveness, we considered whether stac alters Ca^2+^-dependent modulation of Ca_V_2 isoforms that are abundant in the central nervous system. For Ca_V_2.1, CaM elaborates both CDF and CDI (DeMaria et al., 2001; Lee et al., 2000). However, the Ca^2+^-sensitivity of CDI process is over 50-fold weaker than that of CDF, casting this negative feedback beyond physiological bounds (Lee et al., 2015). As such, we probed whether stac tunes CDF of Ca_V_2.1 using a well-established prepulse protocol (DeMaria et al., 2001; Thomas and Lee, 2016). Figure 1G displays wildtype Ca_V_2.1 currents in the absence of stac2. On presentation of an isolated test pulse to 0 mV, the activation of Ca^2+^ current follows a biphasic response (gray trace). Following a brief voltage prepulse, however, the ensuing test pulse yields enhanced Ca^2+^-currents with monophasic activation reflecting CDF (red trace). Further quantification revealed no change in CDF of Ca_V_2.1 following the addition of stac2 in both exemplar current recordings (Figure 1G) and population data (Figure 1H; Figure 1—figure supplement 1D). For Ca_V_2.2, CaM-regulation manifests as a kinetically slow CDI (Figure 1I) (Liang et al., 2003), that persists despite stac co-expression (Figure 1I–J; Figure 1—figure supplement 1E). Here CDI is quantified by metric CDI_800_ = 1 – *r*_Ca_/*r*_Ba_, measured following 800 ms of depolarization. Likewise, neuronal Ca_V_2.3 also possesses robust CDI, which is spared with stac2 co-expression (Figure 1Figure 1K–L; Figure 1—figure supplement 1F).

Lastly, we tested whether stac suppresses Ca^2+^-regulation of Na_V_1, related to Ca_V_1. Although all Na_V_1 possess a CI module homologous to both Ca_V_1 and Ca_V_2 (Babitch, 1990), CDI that bears mechanistic similarity to Ca_V_ has been identified only in Na_V_1.4 (Ben-Johny et al., 2014). Unlike Ca_V_, Na_V_ channels do not convey Ca^2+^ influx that triggers Ca^2+^-feedback. We used rapid photo-uncaging of Ca^2+^ to produce a step-like increase in intracellular [Ca^2+^]_i_, whose magnitude is simultaneously monitored via fluorescent indicators. Figure 1M displays baseline Ca^2+^-regulation of Na_V_1.4. As CDI is kinetically slow in comparison to fast inactivation, we applied a train of step depolarizations evoked at 10 Hz to probe Ca^2+^-dependent effects (Ben-Johny et al., 2014). Without Ca^2+^-uncaging, peak Na_V_1.4 currents remained steady (gray dots). In response to an ~10 μM Ca^2+^ step, the peak Na current declined rapidly revealing CDI (red envelope). Stac overexpression, however, does not disrupt Na_V_1.4 CDI (Figure 1M–N; Figure 1—figure supplement 1G). Overall, these results show the specificity of stac in tuning Ca^2+^-regulation of Ca_V_1 channels.

### Stac interacts with Ca_V_1 CI module to elicit CDI suppression

**Figure 2.**
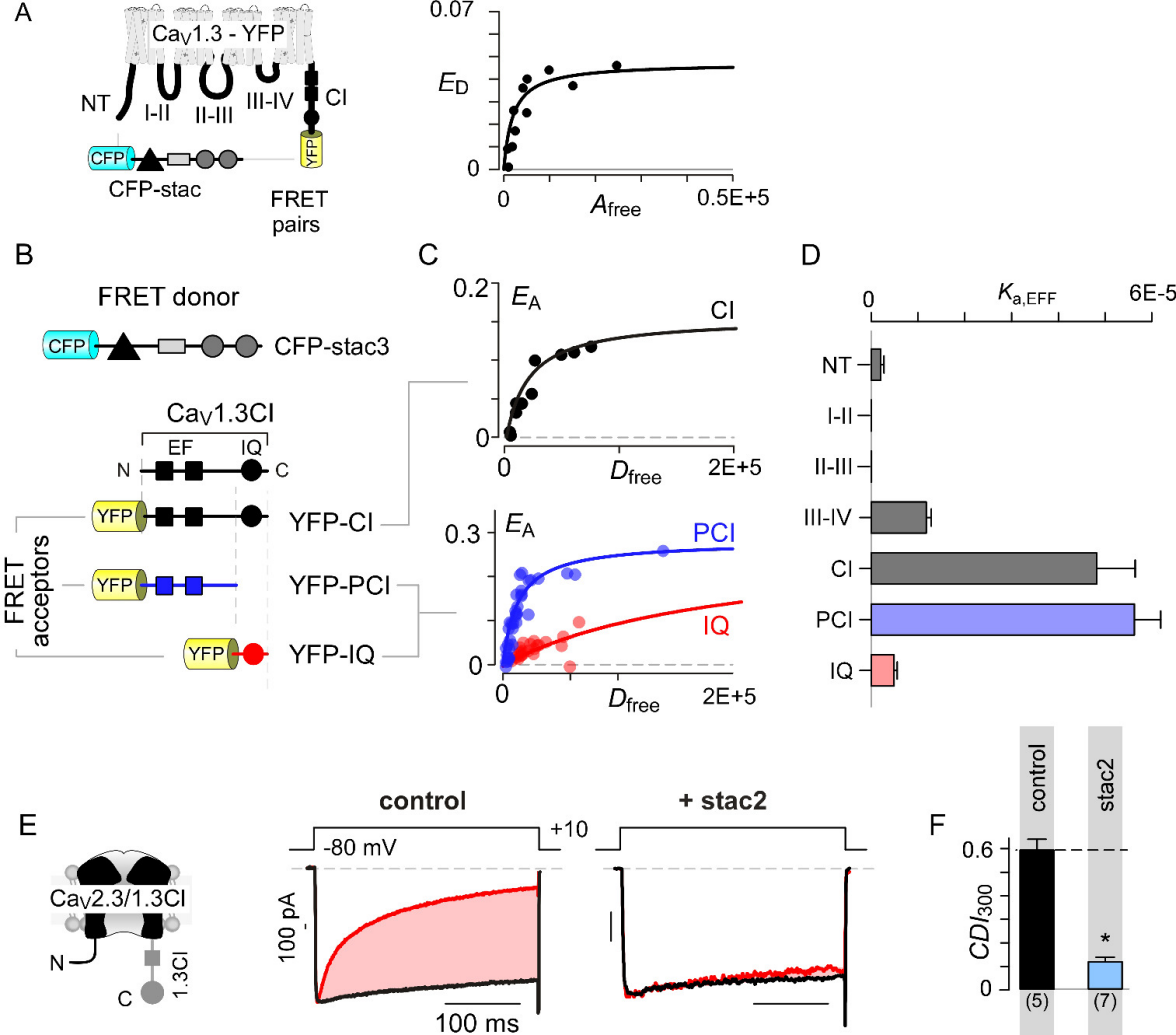
Stac interacts with the channel carboxy-terminus. (**A**) Live-cell FRET 2-hybrid assay shows high-affinity interaction between CFP-tagged stac3 with YFP-tethered holo-Ca_V_1.3 channels in the presence of auxiliary β_2A_ and α_2_δ subunits. (**B**) Cartoon shows FRET pairs, CFP-stac3 with YFP-CI, YFP-PCI, and YFP-IQ of Ca_V_1.3. (**C**) FRET-binding curves show robust binding of stac3 to both the CI and PCI segment while binding to IQ is weaker. (**D**) Bar graph summarizes the relative association constant, *K*_a,EFF_, of stac2 binding to major channel intracellular domains. (**E–F**) Transferring Ca_V_1.3_S_ CI to Ca_V_2.3 (Ca_V_2.3/1.3 CI) unveils latent stac2-mediated suppression of CDI. Format as in Figure 1A – B.

We sought to identify molecular mechanisms that underlie selective Ca_V_1 modulation by stac. As the stac effect here is inferred based on overexpression analysis, we determined relative concentration requirements for stac binding to Ca_V_1 holo-channel complexes within the milieu of living cells. We used live cell FRET 2-hybrid assay (Erickson et al., 2001) to probe the interaction of CFP-tagged stac3 with YFP-linked Ca_V_1.3_S_. As all three stac variants suppress the CDI of all Ca_V_1 isoforms, we chose Ca_V_1.3 as YFP-tethered channels and a repertoire of YFP-tagged intracellular loop peptides are readily available for in-depth binding analysis (Yang et al., 2014). Stac3 was selected for its high potency in suppressing Ca_V_1.3 CDI (Figure 1D). Accordingly, we quantified FRET efficiency (*E*_D_) between FRET pairs co-expressed in individual cells. By leveraging stochastic expression of the FRET pairs in cells, we obtained a saturating Langmuir relationship between *E*_D_ and the free acceptor concentration (*A*_free_) permitting estimation of relative binding affinities (*K*_d,EFF_). Thus probed, we obtained a Ca_V_1.3 holo-channel affinity for stac3 of *K*_d,EFF_ = 1458 ± 251 *D*_free_ units proportional to ~47 nM (Figure 2A). By comparison, similar analysis of CaM binding to Ca_V_1.3 showed *K*_d,EFF_ = 700 *D*_free_units ~ 22 nM (Yang et al., 2014). Consequently, stac’s relatively high binding-affinity for Ca_V_1.3 suggests that it may be a potent modulator even at low concentrations.

**Figure 2—figure supplement 1.**
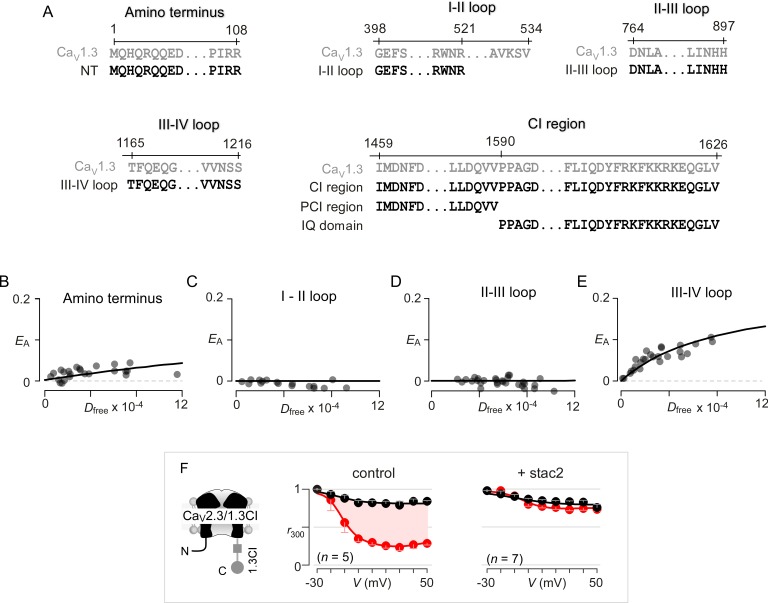
Systematic FRET 2-hybrid scan of major intracellular loops of Ca_V_1.3 with stac. (**A**) Schematic depicts design of YFP-tagged Ca_V_1.3 intracellular loop constructs (NT, I-II loop, II-III loop, III-IV loop, CI region, PCI region, IQ domain). The exact sequence of the N- and C-termini of each peptide as well as locations on the Ca_V_1.3 α subunit are denoted. (**B–E**) FRET 2-hybrid binding curves for stac3 interaction with various channel intracellular loops (black symbol and fit). Each symbol denotes FRET measurements from a single cell. Stac3 binds very weakly to the amino terminal (NT) peptide (**B**) that includes the CaM-binding segment, *NSCaTE*. Both the I-II (**C**) and the II-III loop peptides (**D**) also showed little or no FRET binding. By contrast, the III-IV loop peptide bound appreciably to stac3 (**E**). (**F**) Transfer of CI module of Ca_V_1.3 onto Ca_V_2.3 confers stac modulation. Format as in Figure 1—figure supplement 1A. In the absence of stac2, Ca_V_2.3/1.3 CI exhibits robust CDI triggered by local Ca^2+^ isolated using 10 mM BAPTA (middle subpanel). Stac2 abolishes CDI of Ca_V_2.3/1.3 CI (right subpanel).

With holo-channel binding assured, we systematically scanned YFP-tagged Ca_V_1.3 intracellular domains (Yang et al., 2014) for stac binding sites (Figure 2B; Figure 2—figure supplement 1A). We found that stac3 binds well to the CI region (*K*_d,EFF_ = 20697 ± 3023 *D*_free_~0.67 ± 0.1 μM, Figure 2C). By contrast, analysis of the amino-terminus, intracellular loops between domains I and II (I-II loop), domains II and III (II-III loop), and domains III and IV (III-IV loop) revealed far weaker binding (Figure 2D; Figure 2—figure supplement 1B–E). To further localize the putative binding loci, we subdivided the CI module into two: (1) a proximal CI segment (PCI) composed of dual vestigial EF hand and preIQ segments and (2) the IQ domain (IQ). The YFP-tagged PCI segment bound stac3 with approximately tenfold higher affinity (*K*_d,EFF_ = 17725 ± 3990 *D*_free_~0.58 ± 0.1 μM) than the downstream IQ domain (*K*_d,EFF_ = 204739 ± 25465 *D*_free_~6.67 ± 0.8 μM) (Figure 2C–D). In all, systematic FRET analysis reveals that stac binds to Ca_V_1 CI relying on upstream elements including the dual vestigial EF hand and preIQ domains, an interface distinct from that for CaM (Bazzazi et al., 2013; Minor and Findeisen, 2010).

To test for functional relevance of stac binding to the Ca_V_1 CI module, we sought to confer stac-sensitivity to a stac-insensitive channel via a chimeric approach. We turned to Ca_V_2.3 that lacks strong stac-mediated CDI suppression, yet forms functional chimeras with Ca_V_1 (Mori et al., 2008; Yang et al., 2014). We replaced the CI region of Ca_V_2.3 with the corresponding segment from Ca_V_1.3 (Ca_V_2.3/1.3 CI). Devoid of stac, Ca_V_2.3–1.3 CI channels exhibit CDI isolated by high intracellular buffering (Figure 2E–F; Figure 2—figure supplement 1F). In contrast to wildtype Ca_V_2.3, stac2 co-expression attenuated CDI (p=4.7 × 10^−4^, Figure 2E–F; Figure 2—figure supplement 1F), suggesting that Ca_V_1 CI module is necessary for stac-mediated CDI suppression.

### Stac uses an allosteric mechanism to suppresses CaM signaling

**Figure 3.**
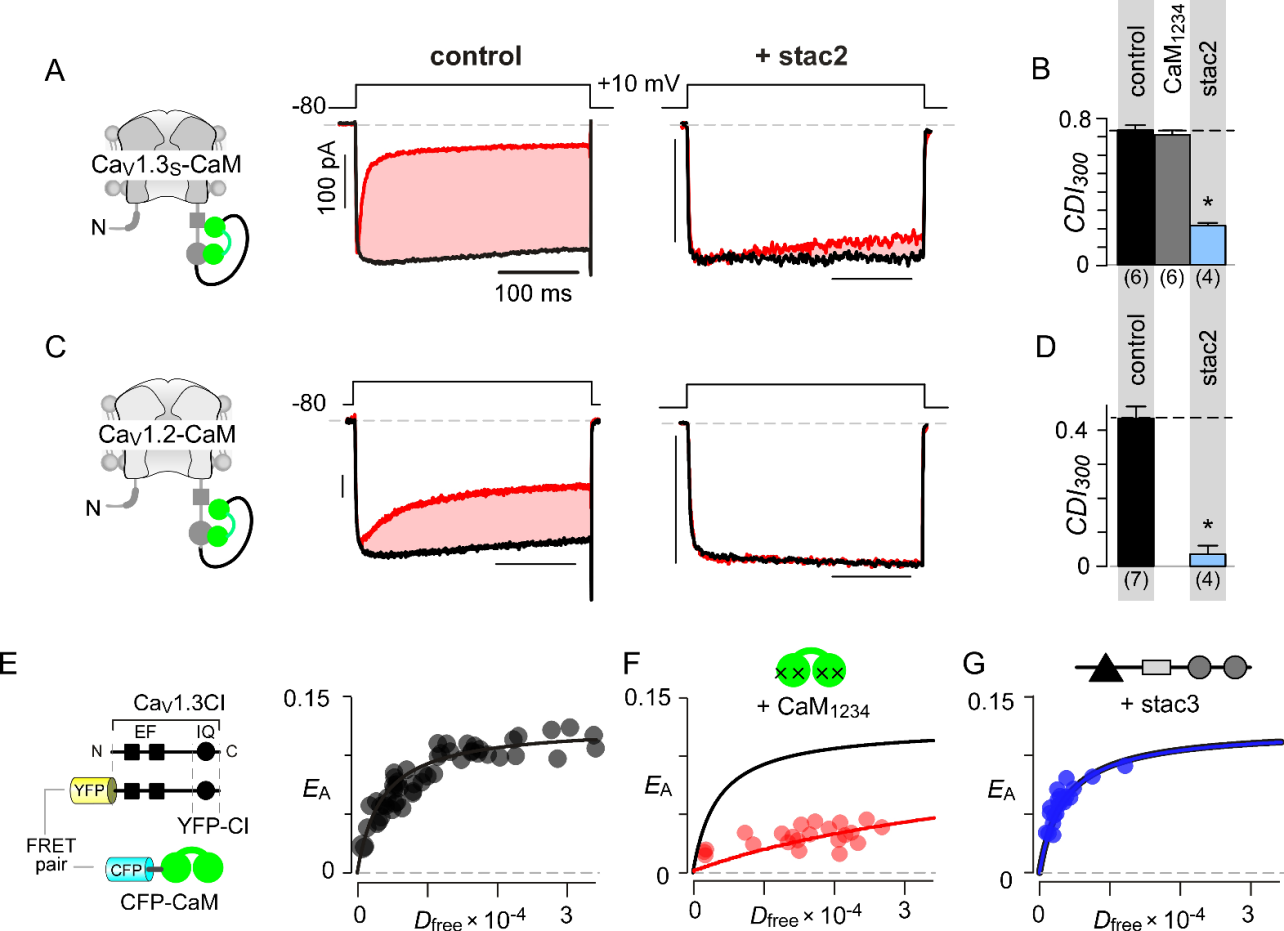
Allosteric regulation of stac by interaction with the channel carboxy-terminus. (**A–B**) Stac2 suppresses CDI of Ca_V_1.3_S_ tethered to CaM. In contrast, fusion of CaM protects Ca_V_1.3_S_ from competitive inhibitors such as CaM_1234_. Format as in Figure 1A–B. (**C–D**) Stac2 suppresses CDI of Ca_V_1.2 tethered to CaM. Format as in Figure 1A–B. (**E**) FRET 2-hybrid assay shows the high-affinity interaction of YFP-tagged Ca_V_1.3 CI to CFP-tagged CaM with relative dissociation constant *K*_d,EFF_ ~4000 ± 291 *D*_free_ units. (**F**) Co-expression of untagged CaM_1234_ with FRET pairs YFP-tagged Ca_V_1.3 CI and CFP-tagged CaM results in a marked reduction in FRET efficiency. (**G**) Co-expression of untagged stac3 spares the binding of YFP-tagged Ca_V_1.3 CI with CFP-tagged CaM, yielding an identical *E*_A_-*D*_free_ relationship to that in the absence of stac3 (**E**).

Given that both CaM and stac share the CI module as an effector site, two disparate mechanistic possibilities may allow suppression of Ca^2+^-regulation. First, stac may competitively displace Ca^2+^-free CaM (apoCaM) from its preassociation site. Second, stac may supersede CaM signaling to the channel pore via an allosteric mechanism. Systematic FRET analysis suggests that stac preferentially binds upstream CI elements (Figure 2D) while high-affinity CaM preassociation is supported via the IQ domain (Bazzazi et al., 2013; Minor and Findeisen, 2010), hinting that the two modulatory proteins may bind concurrently. To rule out competitive displacement of CaM preassociation, we covalently tethered CaM onto the Ca_V_1.3 carboxy-tail using a poly-glycine linker (Ca_V_1.3_S_-CaM) (Mori et al., 2004; Yang et al., 2014). This maneuver preserves CDI (Figure 3A left) and ensures a high local CaM concentration near Ca_V_1 extending into the millimolar range, sufficient to protect the channel from a competitive inhibitor (Mori et al., 2004). Dominant-negative CaM_1234_ with its Ca^2+^-binding sites disabled, typically displaces intact apoCaM from the CI module thereby resulting in a loss of CDI for wildtype channels (Figure 3—figure supplement 1A–B) (Yang et al., 2014). CDI of Ca_V_1.3_S_-CaM persists despite CaM_1234_ co-expression (gray bar, Figure 3B; Figure 3—figure supplement 1C–D). By contrast, CDI of Ca_V_1.3_S_-CaM is diminished by co-expression of stac2 (p=3.8 × 10^−6^, Figure 3A–B; Figure 3—figure supplement 1E) and stac3 (p=4.5 × 10^−4^, Figure 3—figure supplement 1F). As a further test, co-expression of untethered Ca_V_1.3_S_ with both CaM and stac2 also showed low CDI (Figure 3—figure supplement 1G). We observed a similar fate for Ca_V_1.2-CaM with stac2 (p=1.5 × 10^−5^, Figure 3C–D; Figure 3—figure supplement 1H). These findings suggest that stac does not need to dislodge CaM from its Ca_V_1.3 carboxy-tail binding interface to exert functional modulation.

**Figure 3—figure supplement 1.**
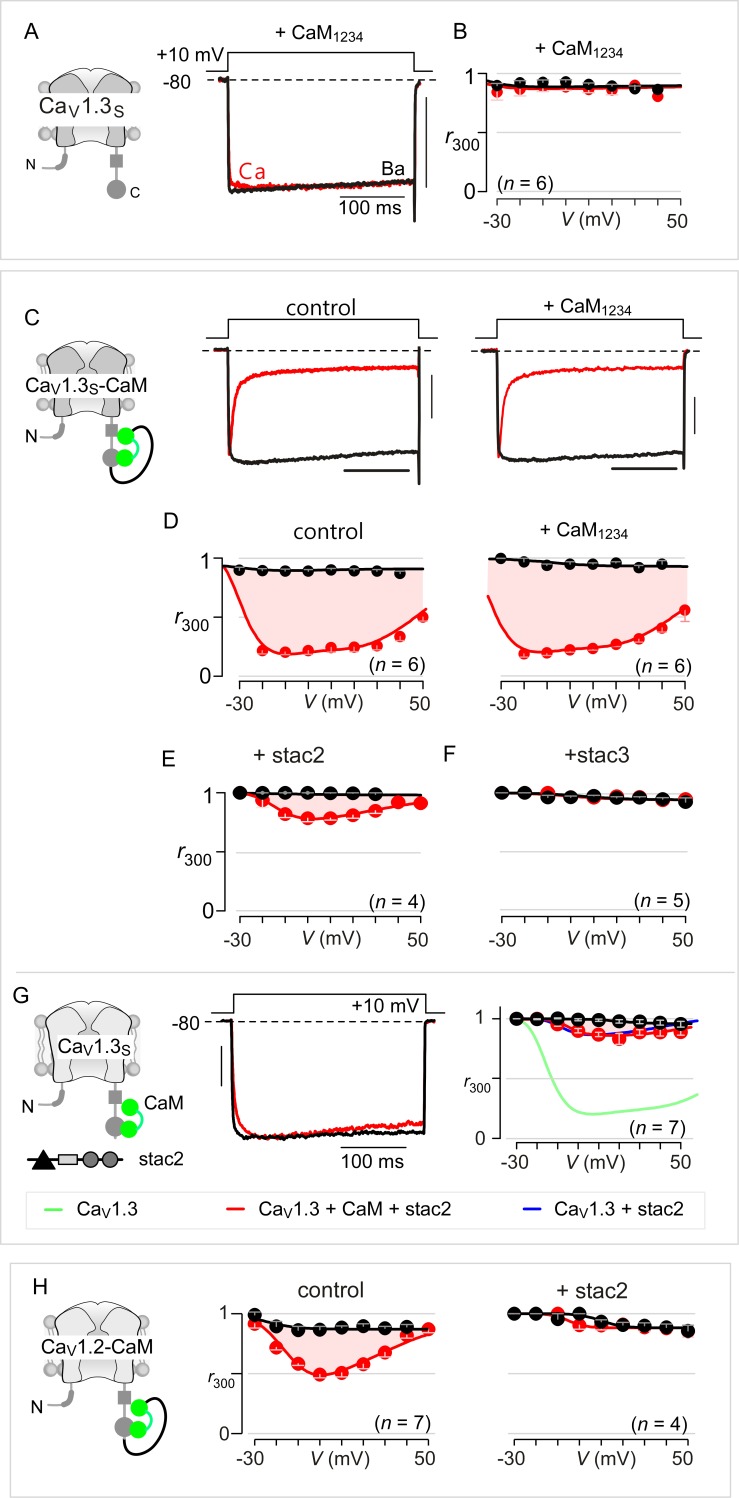
Extended data show that stac acts concurrently with CaM. (**A**) Dominant-negative CaM_1234_ lacking Ca^2+^-binding to all four EF hands abolishes strong CDI of Ca_V_1.3_S_. Left, cartoon schematizes Ca_V_1.3_S_ variant. Right, CDI of Ca_V_1.3_S_ is absent in the presence of CaM_1234_. Here, CaM_1234_ competitively replaces prebound wildtype CaM to suppress CDI. (**B**) Population data of *r*_300_ confirm the abolition of CDI for Ca_V_1.3_S_ upon co-expression of CaM_1234_. Format as in Figure 1—figure supplement 1A. (**C**) Fusion of CaM_WT_ protects Ca_V_1.3_S_ from CaM_1234_, a competitive inhibitor of CDI. Exemplar currents show robust CDI of Ca_V_1.3_S_-CaM under both control conditions and in the presence of CaM_1234_. Control data here reproduced from main text Figure 3A for symmetry. (**D**) Population data of *r*_300_ confirm the presence of CDI for Ca_V_1.3_S_-CaM despite overexpression of CaM_1234_. Format as in Figure 1—figure supplement 1A. These results demonstrate that CaM-fusion can protect Ca_V_1.3 from competitive inhibition of CDI. (**E–F**) Co-expression of stac2 (**E**) and stac3 (**F**) abolishes CDI of Ca_V_1.3_S_-CaM. (**G**) Stac2 diminishes CDI of Ca_V_1.3 despite CaM overexpression. (**H**) Population data of *r*_300_ demonstrate that stac2 abolishes CDI of Ca_V_1.2 with CaM tethered to the carboxy-terminus (Ca_V_1.2-CaM).

To test this possibility, we undertook FRET 2-hybrid assay comparing binding of CFP-tagged CaM to YFP-tagged Ca_V_1.3 CI in the presence and absence of unlabeled stac3. If stac3 were to competitively dislodge CaM, then this binding is predicted to be weakened. At baseline, CaM binds to Ca_V_1.3 CI with a relative dissociation constant, *K*_d,EFF_ ~4000 ± 291 *D*_free_ units (Figure 3E) (Ben Johny et al., 2013). Upon co-expression of CaM_1234_, this baseline binding is weakened ~11 fold, yielding a relative affinity of 47153 ± 4815 *D*_free_ units (Figure 3F). By contrast, co-expression of stac3 did not appreciably perturb CaM binding to the CI module with *K*_d,EFF_ = 4182 ± 330 *D*_free_ units (Figure 3G). These results suggest that both stac and CaM act concurrently via distinct sites on the channel carboxy-tail, in contradiction with a competitive mechanism.

### Elementary mechanisms underlying stac-regulation of Ca_V_1

**Figure 4.**
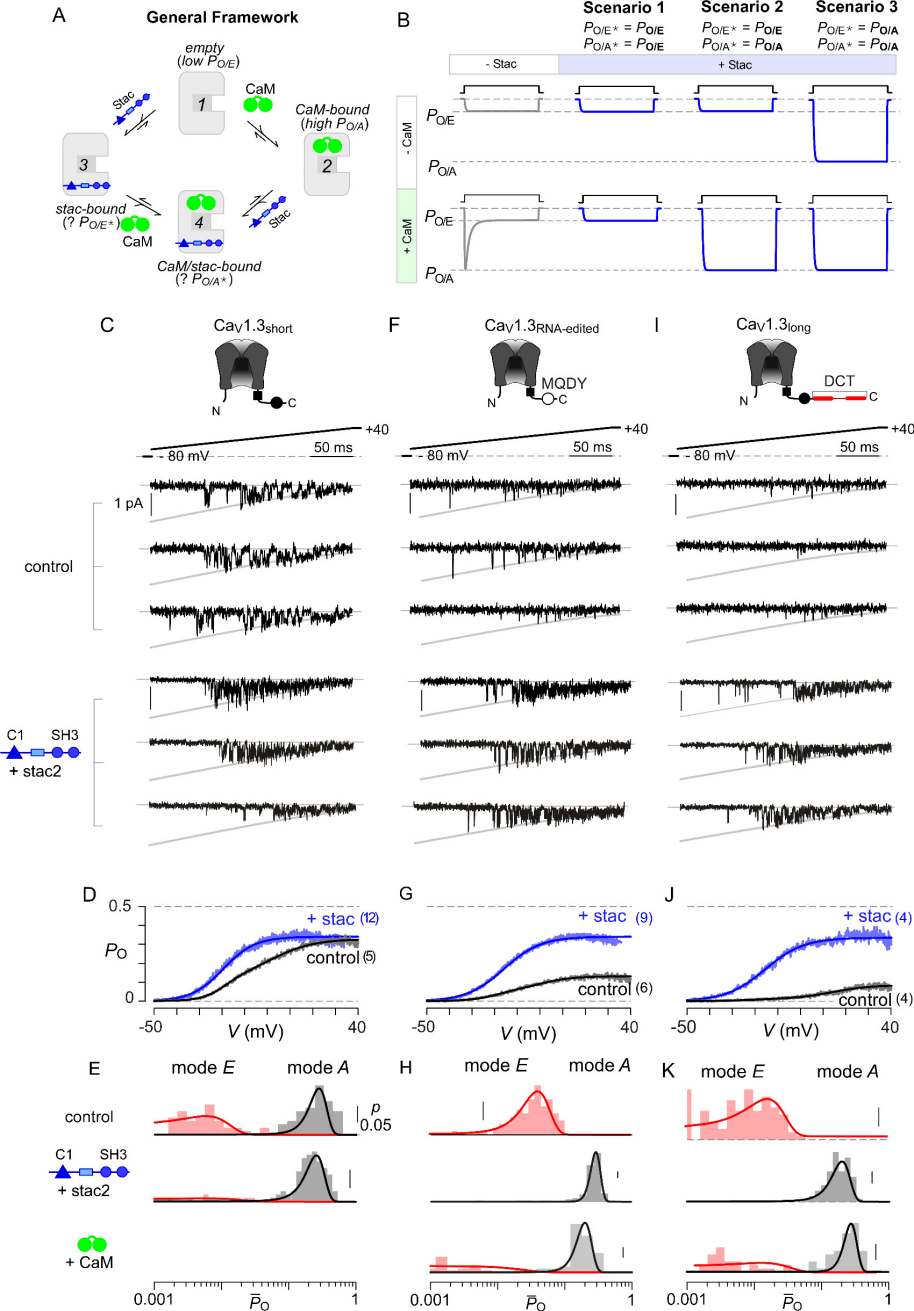
Stac enhances the *P*_O_ of Ca_V_1.3. (**A**) A general four-state scheme for stac and CaM modulation. (1) Ca_V_1.3_S_ devoid of CaM and stac possess a low baseline *P*_O_ (*P*_O/E_). (2) Without stac, apoCaM binding to Ca_V_1.3_S_ upregulates baseline *P*_O_ (*P*_O/A_). Baseline *P*_O_ of Ca_V_1.3_S_ bound to stac in the absence (configuration 3, *P*_O/E*_) and the presence of apoCaM (configuration 4, *P*_O/A*_) are unknown. (**B**) Schematic outlines three mechanistic possibilities for stac binding to Ca_V_1 and their functional outcomes. Scenario 1, stac uniformly suppresses *P*_O_ of Ca_V_1 (*P*_O/E_) and abolishes CDI. Scenario 2, apoCaM tunes baseline *P*_O_ of Ca_V_1 despite concurrent stac binding. Stac, nonetheless, abrogates CDI. Scenario 3, stac uniformly upregulates the baseline *P*_O_ of Ca_V_1 and abolishes CDI (*P*_O/A_). (**C**) Top, cartoon shows the canonical Ca_V_1.3_S_ variant with a high apoCaM binding affinity. Single-channel analysis of recombinant Ca_V_1.3_S_ in the absence (middle) and presence of stac (bottom). In both panels, the unitary Ba^2+^ currents during voltage-ramp are shown between −50 mV and +40 mV (slanted gray lines, GHK fit). Robust Ca_V_1.3 openings are detected in the absence and presence of stac. (**D**) Average single-channel *P*_O_-voltage relationship for Ca_V_1.3_S_ obtained from multiple patches in the absence (gray) and presence of stac2 (blue). Error bars indicate ±S.E.M. for specified number of patches and 80–150 stochastic records per patch. (**E**) Histogram shows distribution of single-trial average *P*_O_ ($\overset{-}{P}$_O_) for the voltage range -30 mV ≤ *V* ≤ +25 mV under control (top), stac-bound (middle), and CaM-bound (bottom) conditions. $\overset{-}{P}$_O_-distribution is bimodal in the absence of stac corresponding to high *P*_O_ (gray) and low *P*_O_ (rose) gating modes. With stac, $\overset{-}{P}$_O_-distribution is largely restricted to the high *P*_O_ mode. (**F–H**) Single-channel analysis of a recombinant Ca_V_1.3_RNA-edited_ variant reveals a marked upregulation in the baseline *P*_O_ in the presence of stac compared with control conditions in which apoCaM preassociation is weak. Absent stac or CaM, single-trial $\overset{-}{P}$_O_-distribution is restricted to the low *P*_O_ limits, With either stac or CaM, the high *P*_O_ gating mode re-emerges. Format as in (**C–E**). (**I–K**) Stac also upregulates the baseline *P*_O_ of Ca_V_1.3_L_, an alternatively spliced variant, by stabilizing the high *P*_O_ gating configuration. Format as in (**C–E**).

Beyond Ca^2+^-dependent regulation, apoCaM binding tunes the baseline activity of Ca_V_ channels (Adams et al., 2014). Absent stac, Ca_V_1 lacking prebound CaM adopts a low *P*_O_ configuration (empty configuration, *P*_O/E_) while apoCaM binding switches channels into a high *P*_O_ mode (CaM-bound configuration, *P*_O/A_) (Adams et al., 2014). Ca^2+^/CaM divests this initial enhancement in *P*_O_ and returns channels into a low *P*_O_ gating mode (*P*_O/E_) manifesting as CDI. The addition of stac as a regulatory agent enriches this modulatory scheme (Figure 4A). Three distinct scenarios may underlie suppression of Ca^2+^-regulation by stac (Figure 4B): (1) stac binding may pre-inhibit channels into the low *P*_O_ configuration (*P*_O/E_) akin to Ca^2+^-inactivated channels and prevent further Ca^2+^-modulation, (2) stac may obstruct Ca^2+^/CaM regulation while allowing apoCaM to change baseline *P*_O_, (3) stac binding may allosterically lock channels into a high *P*_O_ mode irrespective of CaM-binding status. For Scenario 3, it is possible that the baseline *P*_O_ of Ca_V_1.3 with stac may be distinct from that observed with CaM-overexpression. These three scenarios may be distinguished at the single-molecule level by assessing Ca_V_1 *P*_O_ under various CaM-bound conditions using low-noise electrophysiology. We focused on Ca_V_1.3 given the rich assortment of post-transcriptionally modified variants with distinct CaM binding affinities (Bazzazi et al., 2013; Liu et al., 2010; Singh et al., 2008). We focused on three variants, Ca_V_1.3_S_ with high apoCaM affinity, and Ca_V_1.3_MQDY_ and Ca_V_1.3_L_ with low affinities. These variants possess distinct baseline *P*_O_ and CDI and constitute a convenient platform to identify stac-dependent effects (Adams et al., 2014; Tan et al., 2011).

**Figure 4—figure supplement 1.**
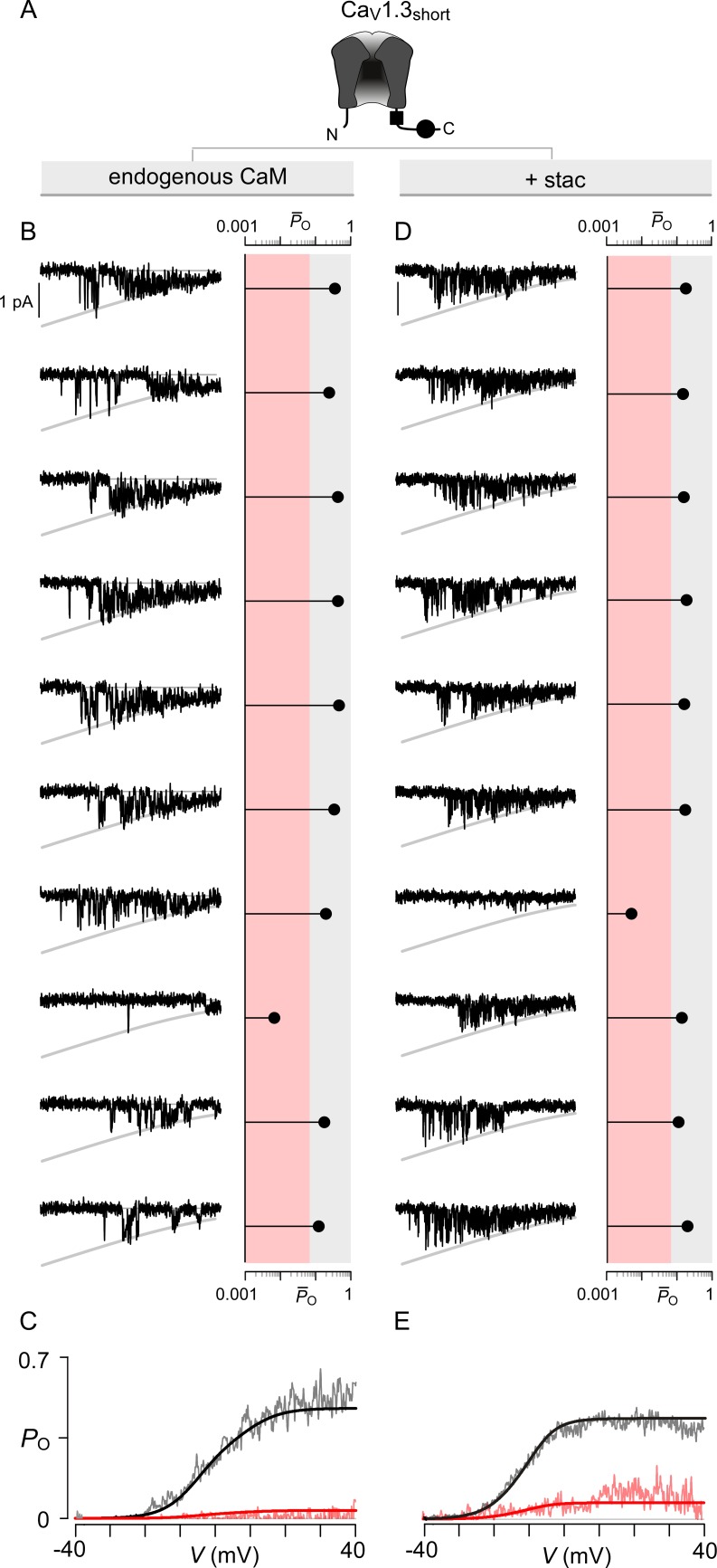
Extended data show that stac2 preferentially biases a high *P*_O_ gating mode for Ca_V_1.3. (**A**) Cartoon schematizes Ca_V_1.3_S_. (**B**) Left, 10 sequential single-channel trials of Ca_V_1.3_S_ under endogenous levels of CaM illustrate high *P*_O_ gating mode with rare sojourns into a low *P*_O_ gating mode. Here, traces show Ba^2+^ currents elicited every 12 s in response to a voltage ramp shown between -40 mV and +40 mV. Right, diary plot displays single trial average *P*_O_ computed for the voltage-range -30 mV ≤ *V* ≤ +25 mV (${\overset{-}{P}}_{O}$ (-30 ≤ *V* ≤ 25)). Dashed line discriminates traces to low *P*_O_ (red shaded area) or high *P*_O_ (gray shaded area) categories as previously established. (**C**) Average *P*_O_ at each voltage calculated for all traces within the high *P*_O_ range (gray region in (**B**)) and the low *P*_O_ range (red shaded region in (**B**)). These relationships estimate the *P*_O_-*V* relationship for high *P*_O_ and low *P*_O_ gating modes. (**D–E**) In the presence of stac2, Ca_V_1.3_S_ preferentially adopts the high *P*_O_ gating configuration. Format as in (**B–C**).

**Figure 4—figure supplement 2.**
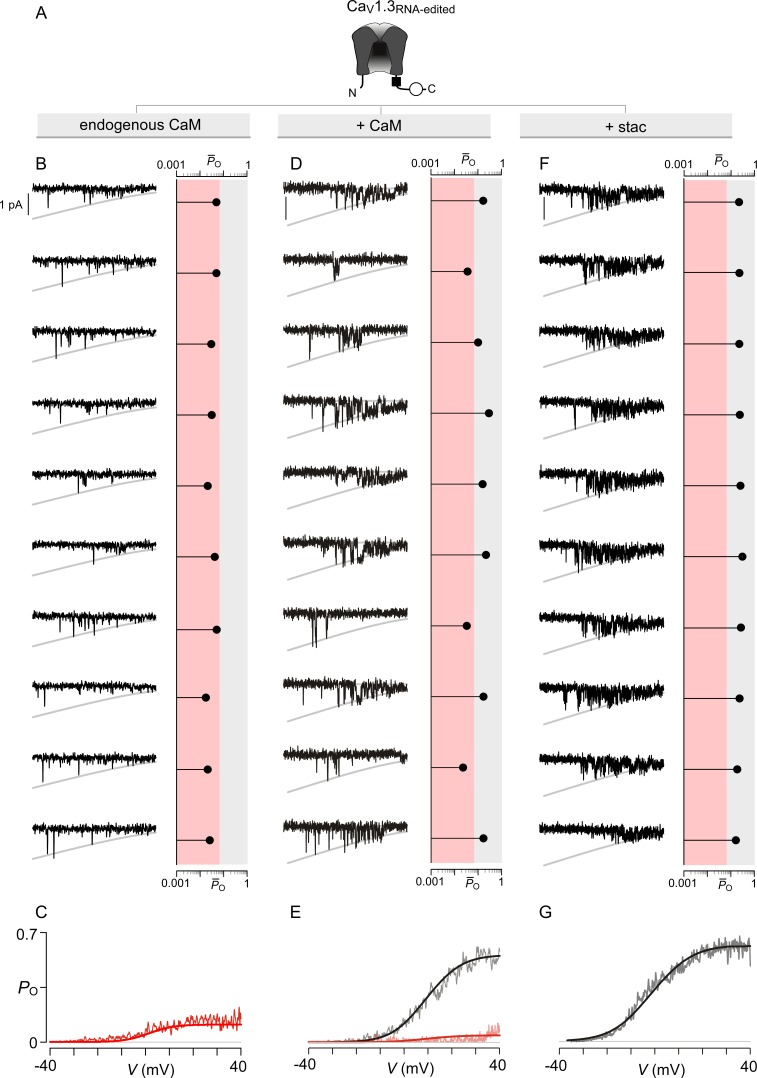
Extended data show that stac2 and CaM enhance the *P*_O_ of Ca_V_1.3_RNA-edited_ variant via discreet transitions into a high *P*_O_ gating mode. (**A**) Cartoon schematizes Ca_V_1.3_RNA-edited_. RNA-editing results in a methionine substitution of the central isoleuicine residue, resulting in sharply diminished CaM binding. Functionally, CaM binding enhances the *P*_O_ of Ca_V_1.3_RNA-edited_. (**B–C**) Under endogenous levels of CaM, Ca_V_1.3_RNA-edited_ exhibits a uniformly low *P*_O_ as evident from individual trials. The average *P*_O_ – *V* relation is consistent with that for the low P_O_ gating mode. Format as in Figure 4—figure supplement 1B–C. (**D–E**) CaM overexpression enhances the *P*_O_ of Ca_V_1.3_RNA-edited_ variant. Exemplar traces depict channels switching between discrete high and low *P*_O_ gating modes. A *P*_O_-*V* relationship for high *P*_O_ and low *P*_O_ gating modes is evident. Format as in Figure 4—figure supplement 1B–C. (**F–G**) Similar to CaM, stac2 overexpression also enhances the *P*_O_ of Ca_V_1.3_RNA-edited_ variant by stabilizing the high *P*_O_ gating mode. Exemplar traces depict channels largely adopting the high gating mode. Format as in Figure 4—figure supplement 1B–C.

**Figure 4—figure supplement 3.**
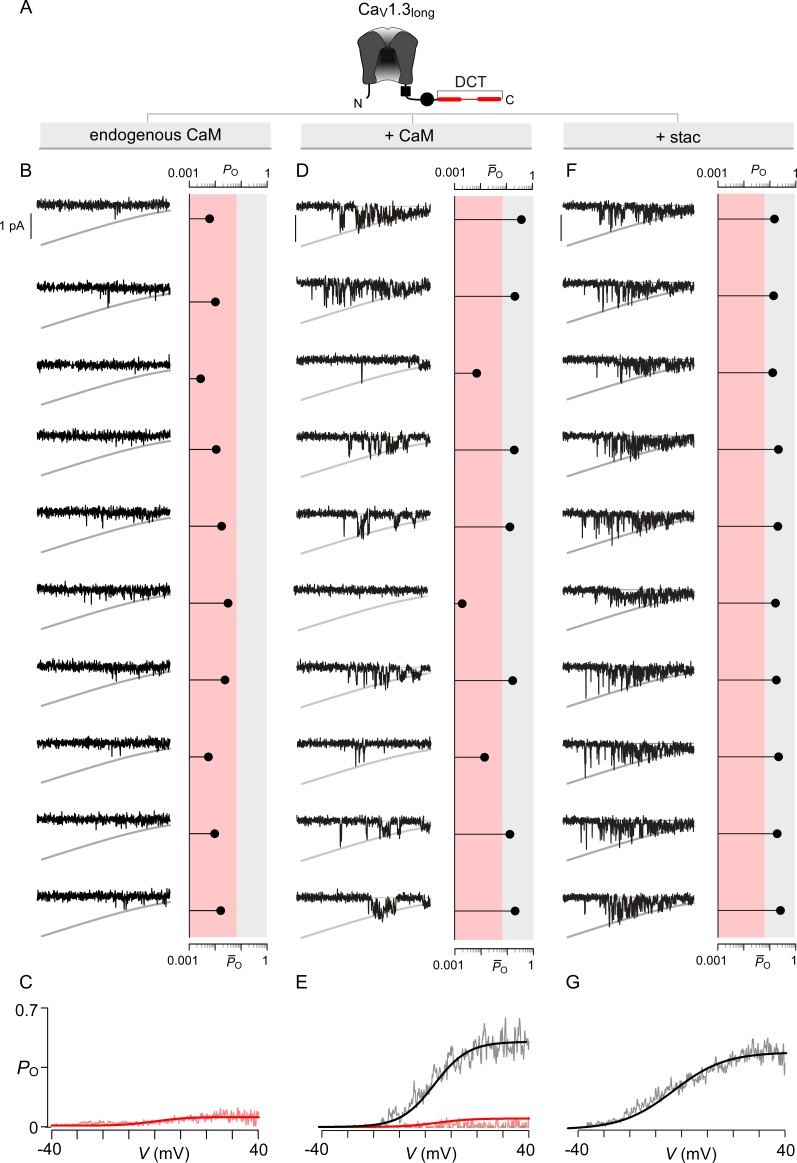
Extended data show that both stac2 and CaM enhance the *P*_O_ of Ca_V_1.3_L_ variant. (**A**) Cartoon schematizes Ca_V_1.3_L_. The long-splice variant includes a distal carboxy-tail (DCT) that interacts with the IQ domain and competitively displaces CaM. (**B–C**) Under endogenous levels of CaM, Ca_V_1.3_L_ exhibits a uniformly low *P*_O_ as evident from individual trials. The average *P*_O_ – *V* relation is consistent with that for the low *P*_O_ gating mode. Format as in Figure 4—figure supplement 1B–C. (**D–E**) CaM overexpression enhances the *P*_O_ of Ca_V_1.3_L_ variant. Exemplar traces depict channels switching between discrete high and low *P*_O_ gating modes. Format as in Figure 4—figure supplement 1B–C. (**F–G**) Similar to CaM, stac2 overexpression also enhances the *P*_O_ of Ca_V_1.3_L_ variant by stabilizing the high *P*_O_ gating mode. Exemplar traces depict channels largely adopting the high gating mode. Format as in Figure 4—figure supplement 1B–C.

First, we analyzed Ca_V_1.3_S_ in the presence and absence of stac (Figure 4C–E) to determine whether stac may promote channel entry into a low *P*_O_ gating configuration. Ca_V_1.3_S_ is typically prebound to CaM at endogenous CaM concentrations given its high affinity (Adams et al., 2014). Ba^2+^ is used as a charge carrier to estimate baseline behavior of channels without confounding effects of CDI. A slow voltage-ramp (shown between −50 and +40 mV) evokes stochastic channel openings that approximate near steady-state *P*_O_ at each voltage. Stochastic records displayed in Figure 4C show channel openings as downward deflections to the open level (gray curves) and closures correspond to the zero-current portions of the trace. Robust openings are detected both in the presence and absence of stac (Figure 4C). To estimate the steady-state *P*_O_ – voltage relationship, we averaged many stochastic records to obtain a mean current that is divided into the open level and averaged over multiple patches. Ca_V_1.3_S_ variant exhibits high *P*_O_ in the absence of stac (Figure 4D) (Adams et al., 2014). Upon stac2 co-expression, the open probability remains high with ~10 mV hyperpolarizing shift in the voltage-dependence of activation (Figure 4D). We scrutinized single-channel trials to assess changes in gating modes. Figure 4—figure supplement 1 displays 10 sequential trials of Ca_V_1.3 single-channel activity evoked by voltage-ramps introduced at 12 s intervals both in the presence and absence of stac. In the absence of stac, Ca_V_1.3_S_ activity switches between epochs of high and low activity, as evident from the diary plot of average *P*_O_ within individual trials (${\bar{P}}_{o}$) (Figure 4—figure supplement 1B). Analysis of single-trial ${\bar{P}}_{o}$ distribution reveals a bimodal distribution denoting discrete high and low *P*_O_ gating modes (Figure 4E). Upon stac overexpression, channel activity is high as evident from ${\bar{P}}_{o}$-diary plots (Figure 4—figure supplement 1D) and single-trial ${\bar{P}}_{o}$ distribution (Figure 4E). In contradiction with Scenario 1, stac-bound channels are not pre-inhibited; rather, channels preferentially adopt a high *P*_O_ mode.

To distinguish between the second and third mechanistic possibilities, we considered Ca_V_1.3 variants with weakened apoCaM binding affinity that largely reside in the low *P*_O_ configuration (Adams et al., 2014). Accordingly, we tested the baseline *P*_O_ of Ca_V_1.3_MQDY_, an RNA-edited variant whose central isoleucine within the IQ domain is substituted to a methionine, (Bazzazi et al., 2013; Huang et al., 2012) and an alternative splice variant Ca_V_1.3_L_ containing an autoinhibitory domain that competitively displaces CaM (Liu et al., 2010; Singh et al., 2008). In the absence of stac and under endogenous CaM levels, both Ca_V_1.3_MQDY_ (Figure 4F–G) and Ca_V_1.3_L_ (Figure 4I–J) open sparsely, exhibiting a diminished maximal *P*_O_ consistent with channels lacking CaM (Adams et al., 2014; Bock et al., 2011). Indeed, single-channel trials of Ca_V_1.3_MQDY_ (Figure 4—figure supplement 2A–C) and Ca_V_1.3_L_ (Figure 4—figure supplement 3A–C) under endogenous levels of CaM reveal uniformly low activity, with single-trial *P̄*_O_ distribution restricted to low limits (Figure 4H for Ca_V_1.3_MQDY_; Figure 4K for Ca_V_1.3_L_). CaM overexpression with both channel variants reveals the resurgence of epochs of high activity (Figure 4—figure supplement 2D–E; Figure 4—figure supplement 3D–E) and a bimodal *P̄*_O_ distribution with a substantial fraction of trials corresponding to a high *P*_O_ configuration (Figure 4H and K for Ca_V_1.3_MQDY_ and Ca_V_1.3_L_ respectively). Upon stac co-expression, robust channel openings re-emerge for both Ca_V_1.3_MQDY_ (Figure 4F–G) and Ca_V_1.3_L_ (Figure 4I–J) yielding an enhanced baseline *P*_O_ akin to Ca_V_1.3_S_ variant (Adams et al., 2014). Scrutiny of single-channel trials for both channel variants reveal uniformly high channel activity (Figure 4—figure supplement 2F–G for Ca_V_1.3_MQDY_; Figure 4—figure supplement 3F–G for Ca_V_1.3_L_) and single-trial *P̄*_O_ distributions are now within the high activity limits (Figure 4H and K) reminiscent of the CaM overexpression. The steady-state *P*_O_-*V* relations for Ca_V_1.3_S_, Ca_V_1.3_MQDY_, and Ca_V_1.3_L_ in the presence of stac closely approximate each other (Figures 4D, G and J). These findings demonstrate that consistent with Scenario 3, stac-binding locks Ca_V_1.3 channels in the high *P*_O_ configuration and effectively decouples the channel pore from CaM-dependent conformational changes. Moreover, these results highlight the dominance of stac over CaM in Ca_V_1 modulation.

### U-domain constitutes a minimal motif for Ca_V_1 CDI suppression

**Figure 5.**
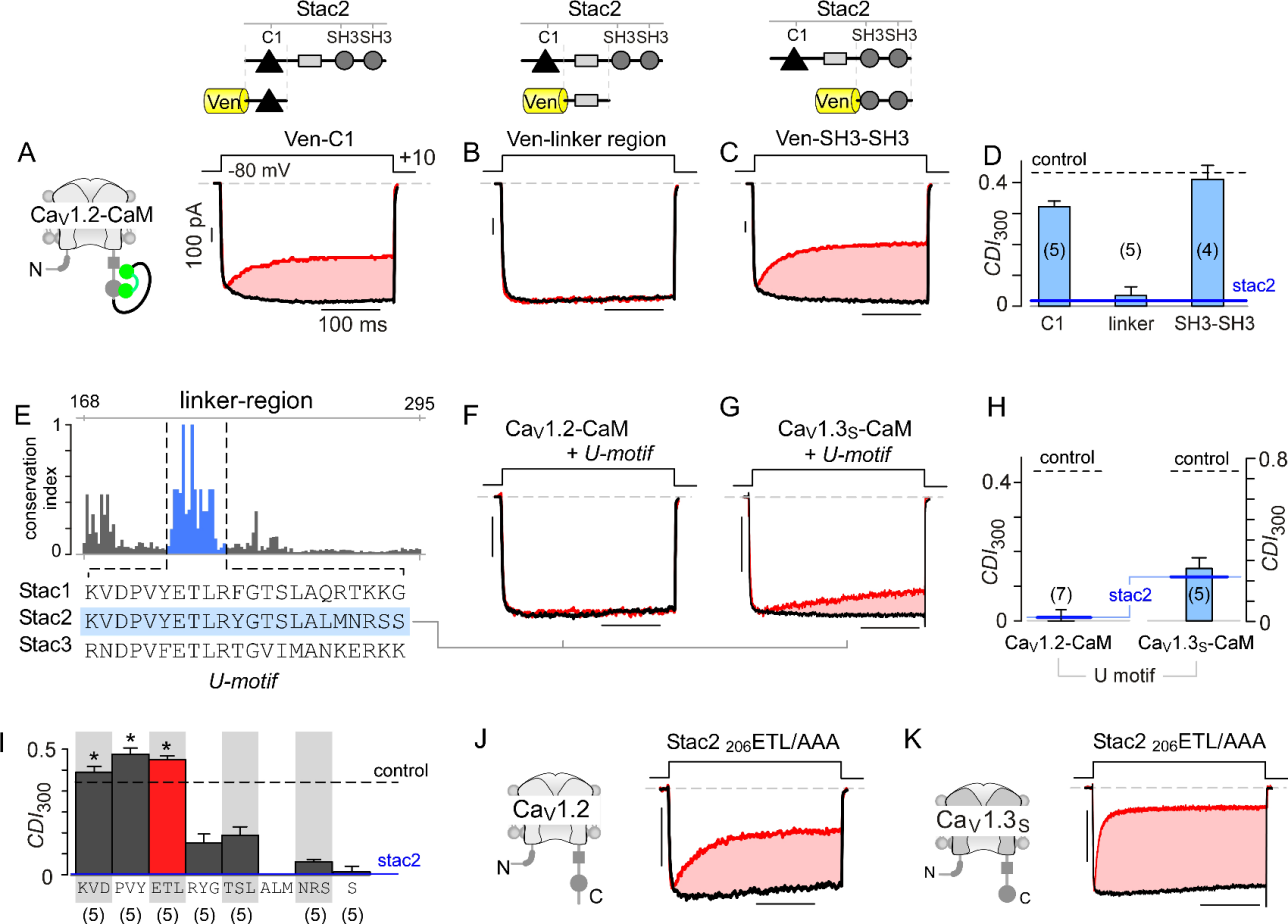
Stac U-domain is a minimal effect domain for suppression of Ca_V_1 CDI. (**A–C**) To localize an effector motif for stac2, CDI of Ca_V_1.2-CaM was quantified in the presence of three stac subdomains: (1) C1, (2) linker region, and (3) SH3-SH3. Exemplar traces in response to a +10 mV voltage-step depolarization show robust CDI of Ca_V_1.2-CaM in the presence of C1 (**A**), and SH3-SH3 (**C**) domains. Co-expression of the linker-region is sufficient to suppress CDI of Ca_V_1.2-CaM (**B**). Format as in Figure 1A. (**D**) Bar graph summarizes population data for Ca_V_1.2-CaM CDI in the presence of the three stac subdomains. Each bar, mean ±S.E.M of CDI_300_ at +10 mV from specified number of cells. CDI levels in the presence (solid blue line) and absence (dashed gray line) of full-length stac2 is reproduced for comparison. (**E**) Bar graph shows degree of conservation for the linker region across 770 orthologs of stac2. A well conserved subsegment termed U-domain is shaded blue. (**F–G**) Co-expression of U-domain is sufficient to abolish CDI of Ca_V_1.2-CaM (**F**) and Ca_V_1.3-CaM (**G**). Format as in Figure 1A. (**H**) Bar graph displays population data for CDI of Ca_V_1.2-CaM and Ca_V_1.3-CaM in the presence of U-domain. Each bar, mean ±S.E.M of CDI_300_ at +10 mV from specified number of cells. Dashed line, baseline CDI for both channels in the absence of stac2. Blue line, CDI of both channels in the presence of full-length stac2. (**I**) Systematic alanine scanning mutagenesis of the U-domain reveals critical determinants for stac-mediated suppression of Ca_V_1.2 CDI. For comparison, Ca_V_1.2 CDI in the presence (blue line) and absence (black dashed line) of stac2 are shown. Stac2 mutants _200_KVD/AAA, _203_PVY/AAA, _206_ETL/AAA fully abolish stac2-mediated CDI suppression. (**J**) Exemplar currents show that stac2 mutant _206_ETL/AAA eliminates stac’s ability to suppress Ca_V_1.2 CDI. Format as in Figure 1A. (**K**) Stac2 _206_ETL/AAA also fails to inhibit CDI of Ca_V_1.3_S_. Format as in Figure 1A.

With elementary mechanisms discerned, we turned to identify stac motifs functionally critical for allosteric suppression of CaM-regulation. Structurally, stac isoforms share a modular architecture composed of a C1 domain linked to two SH3 domains via a largely unstructured linker segment (U-linker region) (Suzuki et al., 1996). As these modular subcomponents typically recognize distinct ligands, we reasoned that their molecular functions may be separable (Cohen et al., 1995; Colon-Gonzalez and Kazanietz, 2006). We trisected stac2 to assess whether individual subcomponents can recapitulate functional regulation. We focused initially on C1 and tandem SH3 domains as these segments were recently shown to be critical for Ca_V_1.1 binding and triadic localization in skeletal muscle (Campiglio and Flucher, 2017; Wong King Yuen et al., 2017). Co-expression of either segment, however, only minimally perturbed CDI of Ca_V_1.2-CaM (Figures 5A, C and D; Figure 5—figure supplement 1A–1C). By contrast, the linker region by itself fully abolished CDI of these channels (p=8.9 × 10^−6^, Figure 5B and D; Figure 5—figure supplement 1D), recapitulating the effect of stac2 on Ca_V_1.2.

**Figure 5—figure supplement 1.**
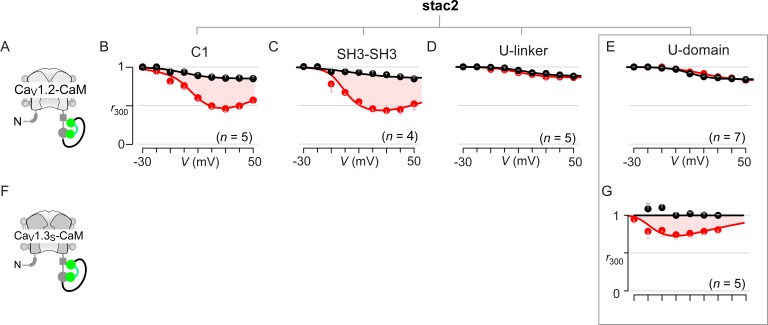
Extended data demonstrate that the U-motif is a minimal domain for suppressing CDI of Ca_V_1.2 and Ca_V_1.3. (**A–C**) Population data confirm that co-expression of either the cysteine-rich domain (C1; **B**) or dual SH3 domains (SH3-SH3; **C**) of stac2 in isolation spares CDI of Ca_V_1.2-CaM (cartoon in **A**). Format as in Figure 1—figure supplement 1A. Each symbol, mean ±S.E.M. of *r*_300_ values obtained from the specified number of cells (n). Here, tethered CaM was used to protect from potential competitive displacement of CaM by stac2 subdomains. (**D–E**) Population data confirm that the U-linker that connects the C1 and dual SH3 domains is sufficient to suppress CDI of Ca_V_1.2-CaM, fully recapitulating the effect of stac on Ca_V_1 channels (**D**). The well-conserved minimal U-motif from stac2 is also sufficient to fully suppress CDI of Ca_V_1.2-CaM (**E**). Format as in Figure 1—figure supplement 1A. Each symbol, mean ±S.E.M. of *r*_300_ values. (**F**) Cartoon shows Ca_V_1.3_S_-CaM. (**G**) Stac2 U-domain also diminished CDI of Ca_V_1.3_S_-CaM as evident from population data of *r*_300_ values. Residual CDI here (red shaded area) is reminiscent of that observed with full-length stac2 and Ca_V_1.3_S_-CaM (Figure 3—figure supplement 1E).

**Figure 5—figure supplement 2.**
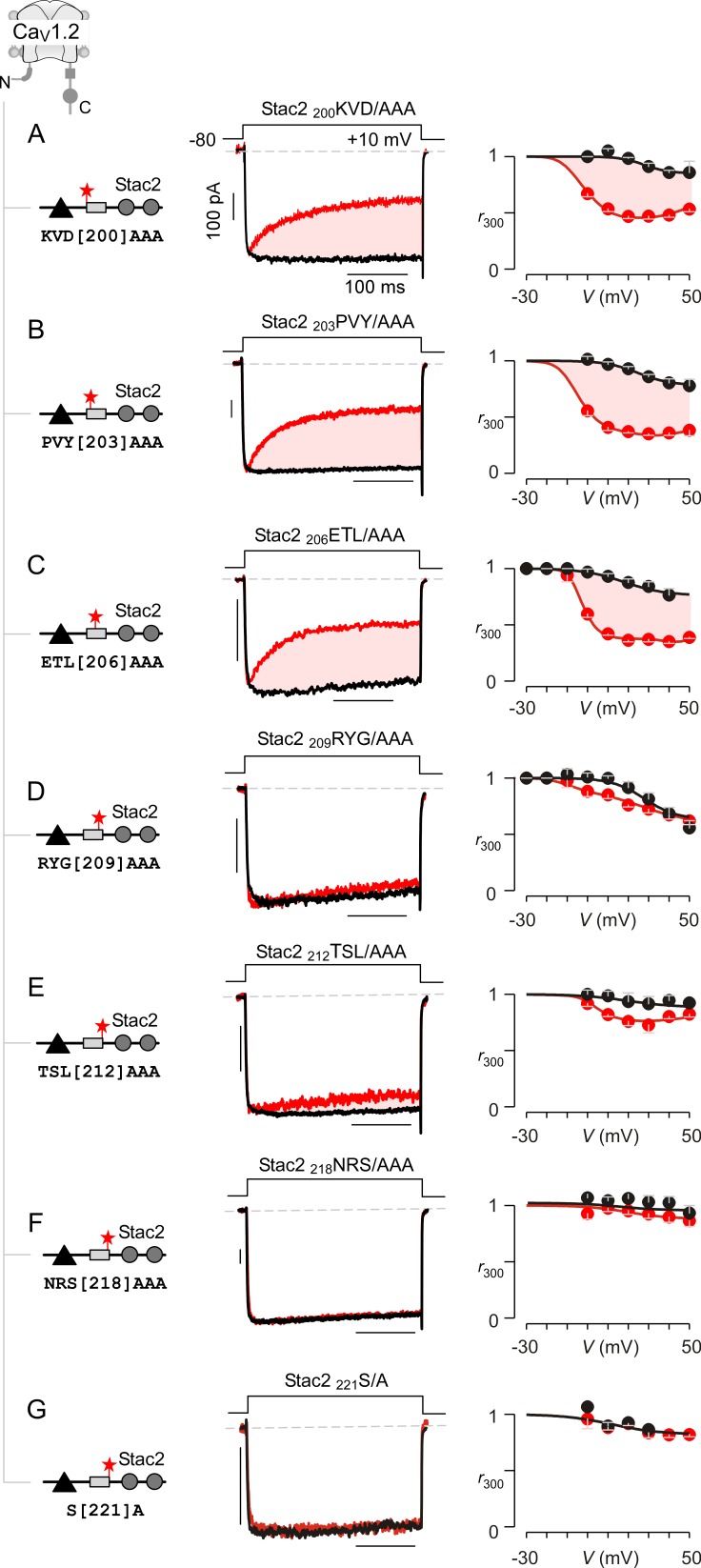
Systematic alanine scanning mutagenesis of minimal U-motif reveals structural determinants for stac modulation of Ca_V_1. (**A**) Triple alanine substitution of residues _200_KVD_202_ abolishes stac modulation of Ca_V_1.2. In comparison to stac2 that fully suppresses CDI of Ca_V_1.2, co-expression of stac2 _200_KVD/AAA spares strong CDI of Ca_V_1.2 suggesting that residues _200_KVD_202_are critical for stac modulation. Left, cartoon schematizes the location of _200_KVD/AAA mutation on full-length stac2. Middle, exemplar current traces show robust CDI of Ca_V_1.2 in the presence of stac2 _200_KVD/AAA. Right, population data showing *r*_300_ values as a function of voltage for Ca^2+^ (red) and Ba^2+^ currents (black). Each symbol, mean ±S.E.M. from five cells. (**B–C**) Triple alanine substitution of stac2 residues _203_PVY_205_ and _206_ETL_208_ abolishes stac modulation of Ca_V_1.2 as evident from strong CDI present despite overexpression of mutant stac. Format as in (**A**). (**D–G**) Ca_V_1.2 CDI is suppressed by stac2 despite alanine substitution of residues, _209_RYG_211_**D**), _212_TSL_214_ (**E**), _218_NRS_220_(**F**), and _221_S (**G**), suggesting that these residues are not necessary for stac modulation of Ca_V_1 channels. Format as in (**A**).

To localize functional segments within the U-linker, we undertook bioinformatic analysis to identify highly conserved regions. We performed multiple sequence alignment of complete sequences of 770 stac orthologs using the MUSCLE algorithm (Edgar, 2004) and subsequently computed an empirical measure for the degree of protein sequence conservation at each position. The degree of conservation is defined as the likelihood of observing the consensus residue at each sequence position divided by the number of distinct residues observed at this position. By this algorithm, perfectly conserved residues will yield a unitary value, whereas poorly conserved residues will have a lower score. We identified a 22-amino acid stretch, termed the U-domain (‘unknown’ domain), exhibiting high conservation (Figure 5E, blue shaded region). Co-expression of U-domain diminishes CDI of both Ca_V_1.2-CaM and Ca_V_1.3-CaM (Figure 5F–H, Figure 5—figure supplement 1E–G). Thus informed, we undertook systematic alanine scanning mutagenesis of the stac2 U-domain to identify key residues (Figure 5I; Figure 5—figure supplement 2). Co-expression of mutant stac2 with triple alanine substitution of residues ETL[206-208] resulted in minimal disruption of Ca_V_1.2 and Ca_V_1.3 CDI (Figure 5J–K), suggesting that these residues are necessary. Further analysis revealed residues PVY[203-205] and KVD[200-202] to be critical for stac function (Figure 5I; Figure 5—figure supplement 2A–2C). Residues outside these loci minimally affected stac modulation of Ca_V_1 (Figure 5I; Figure 5—figure supplement 2D–G). These findings confirm the necessity and sufficiency of U-domain as a minimal motif for preventing CaM-regulation of Ca_V_1.

### U-domain modulates native Ca_V_1 and reshapes cardiac action potentials

Having identified a minimal U-domain for CDI suppression, we sought to assess potential physiological consequences of stac regulation in cardiac myocytes. As stac expression is yet to be identified in myocytes, we first probed its presence using immunohistochemistry with stac1- and stac2-specific antibodies. To ensure that the two antibodies reliably probe the two isoforms, we first evaluated the ability to detect stac isoforms exogenously expressed in HEK293 cells (Figure 6—figure supplement 1). Untransfected cells show minimal stac1 and stac2 immunostaining (Figure 6—figure supplement 1A–B), as confirmed by confocal imaging and population data. By contrast, immunostaining with stac1 antibody shows labeling with cells expressing stac1 but not stac2 or stac3. Similarly, labeling with stac2 antibody reveals substantial fluorescence (*F* > 300) in cells transfected with stac2 but not stac1 or stac3. Thus informed, we assessed expression and localization of stac isoforms in cardiac myocytes (Figure 6—figure supplement 1C–F). Analysis of acutely dissociated adult guinea pig ventricular myocytes (aGPVMs) showed stac2 labeling but not stac1 (Figure 6—figure supplement 1C). Consistent with these findings, immunoblotting with stac2 antibody showed ~50 kDa signal in stac2-transfected HEK293 cells but absent from untransfected cells, confirming the ability of the antibody to detect stac2 (Figure 6—figure supplement 1G). Analysis of aGPVM lysate revealed likely endogenous stac2 with a similar molecular weight to that of recombinant stac2 in HEK293 cells (Figure 6—figure supplement 1G).

**Figure 6.**
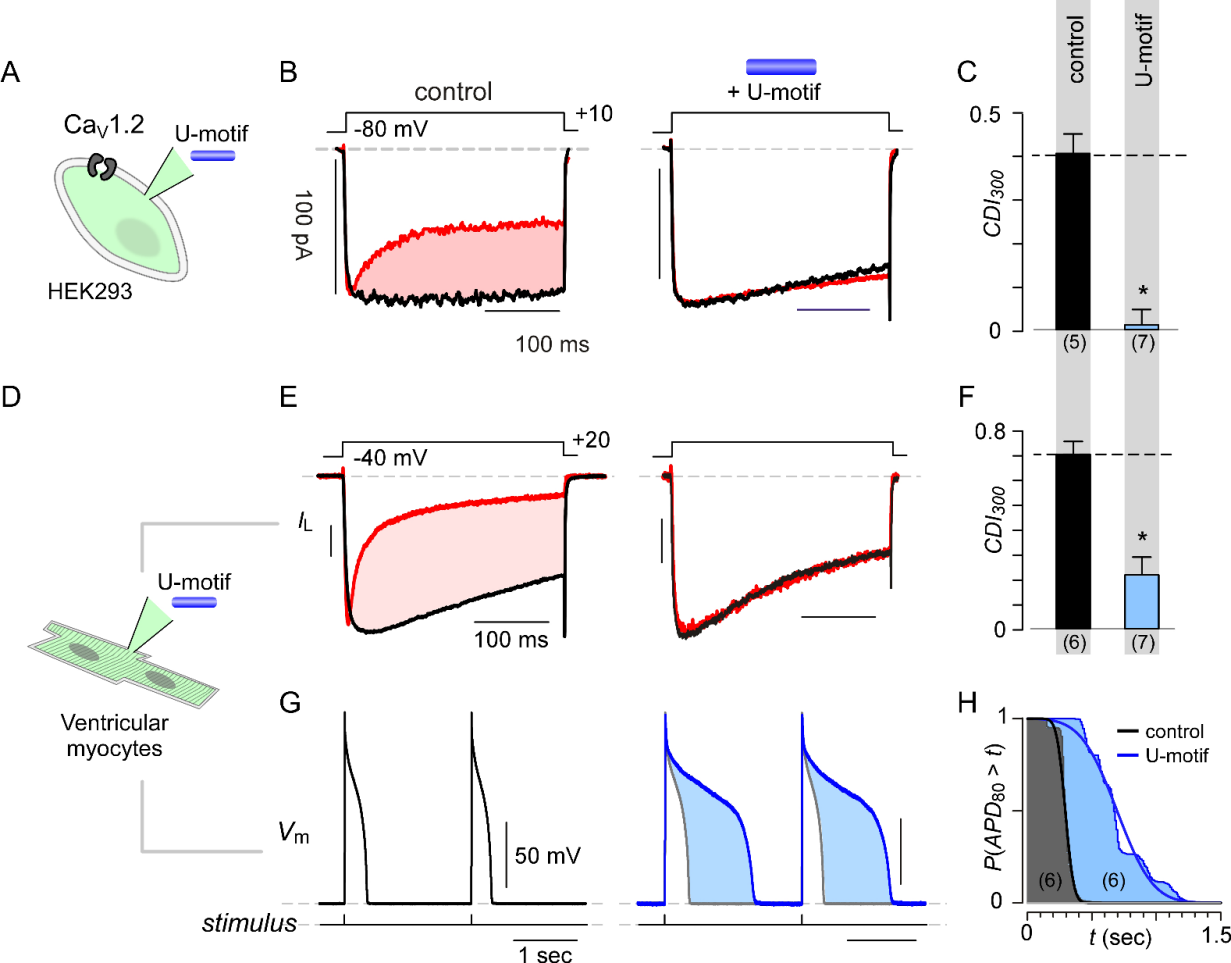
Synthetic U-domain peptide is sufficient for physiological perturbations. (**A**) Schematic illustrates pipette dialysis of custom synthesized U-domain peptide in Ca_V_1.2 heterologously expressed in HEK293 cells, a strategy that emulates acute elevation of cytosolic stac2 levels. (**B–C**) Exemplar traces and population data confirm that pipette dialysis of U-domain suppresses CDI of recombinant Ca_V_1.2 in HEK293 cells. Format as in Figure 1A–B. Control relation in (**C**) is duplicated from Figure 1B. (**D–F**) Pipette dialysis of U-domain abolishes CDI of endogenous L-type current in freshly dissociated ventricular myocytes from adult guinea pigs as evident from exemplar traces and bar graph summary of population data. To eliminate T-type current, the cells were depolarized to −40 mV for a period of 100 ms. Format as in (**A-–C**). (**G**) Exemplar action potential traces of aGPVMs paced at 0.5 Hz with (blue) and without (black) 0.5 μM U-domain in the internal solution. In the presence of U-domain, the action potentials are markedly prolonged (blue shaded area) consistent with a loss of CDI of native L-type current. (**H**) Complement of cumulative distribution (*P*(*APD*_80_ >*t*) of action potential durations (*APD*_80_) obtained in the presence (blue) and absence (black) of U-domain in the internal solution.

Given this baseline expression, we next considered potential effects of fluctuations in ambient stac levels. We synthesized the U-domain of stac2 as a peptide and delivered it via pipette dialysis to acutely elevate the myocyte’s cytosolic concentration. We validated the synthesized peptide by testing its effect on recombinant Ca_V_1.2 expressed in HEK293 cells (Figure 6A). Following pipette dialysis of the U-peptide, CDI of Ca_V_1.2 was reduced as evident from exemplar currents and population data (Figure 6B–C; Figure 6—figure supplement 2A–B). Thus affirmed, we isolated ventricular myocytes from adult guinea pigs (aGPVMs) to probe changes in CDI of native Ca_V_ channels and action potential duration in response to changes in stac levels (Figure 6D). Devoid of U-domain peptide, endogenous Ca^2+^ currents in ventricular myocytes displayed CDI, establishing baseline levels of CaM-regulation (Figure 6E, Figure 6—figure supplement 2D). Pipette dialysis of U-peptide reduced CDI in myocytes (Figure 6E–F, Figure 6—figure supplement 2E). The reduction in overall inactivation of Ca^2+^ currents suggest that fluctuations in stac levels may markedly alter action potential waveforms. To test this possibility, we obtained current-clamp recordings of aGPVMs and compared action potential waveforms in the presence and absence of U-peptide. Figure 6G shows typical voltage profiles of action potentials in aGPVMs paced at 0.5 Hz. Waveforms are stable between traces and the mean action potential duration (*APD*_80_), the duration of time when the action potential is at least 80% of its peak voltage, is 277.9 ± 31.37 ms (mean ±S.E.M., n = 6). Figure 6H displays the complement of the cumulative distribution of *APD*_80_. When the peptide is added to the internal solution, *APD*_80_ is enhanced to 740.1 ± 105.49 ms (n = 6) (Figure 6G–H). Thus, the U-peptide both alters the CDI of endogenous cardiac Ca_V_1, prolongs APD, and may ultimately destabilize rhythmicity of the heart.

**Figure 6—figure supplement 1.**
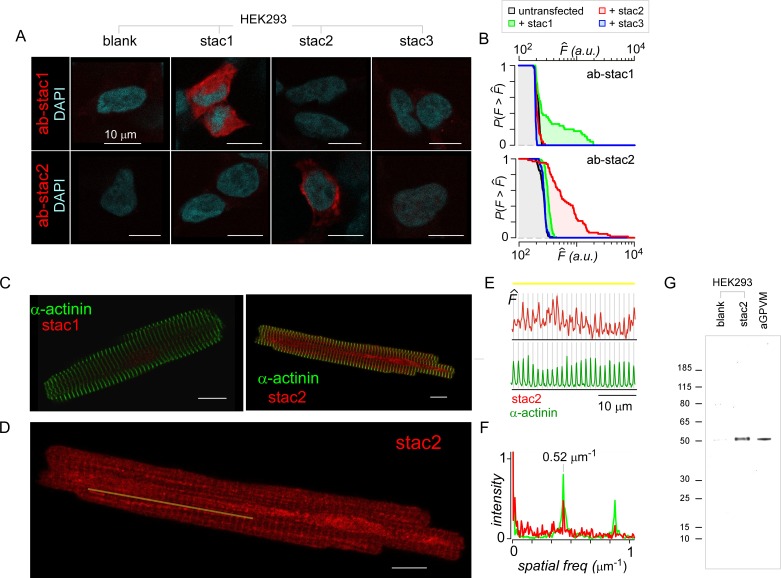
Baseline expression of stac in cardiac myocytes. (**A**) HEK293 transfected with stac1-3 (top) and immunostained with DAPI (cyan) and stac1 (red, top) or stac2 (red, bottom) antibodies confirm selectivity of stac antibodies. (**B**) Population analysis shows the complement of the cumulative distribution of fluorescence intensities ($P(F>\hat{F})$) from 80 to 100 individual HEK293 cells stained with either stac1 (top) or stac2 (bottom) antibodies. Black line, untransfected cells. Data from cells transfected with recombinant stac1, stac2, or stac3 are shown in green, red, and blue, respectively. When probed with stac1 antibody, only cells transfected with exogenous stac1 show appreciable fluorescence above background. Similarly, when probed with stac2 antibody, only cells transfected with exogenous stac2 show fluorescence intensities above background level. (**C**) Acutely dissociated aGPVM immunostained with α-actinin (green) and stac1 (red, left) and stac2 (red, right). Images representative of three fields of views for stac1 and seven fields of view for stac2. (**D**) Stac2 immunostaining reveals weak t-tubular localization in aGPVM. Image reproduced from panel C. Yellow line, line-scan used in (**E**). (**E**) Fluorescence intensity of stac2 (red, top) and α-actinin (green, bottom) across the line-scan shown in (**D**) from left to right. Vertical lines correspond to location of the peak fluorescence intensity for α-actinin. (**F**) Spatial Fourier transform of line scans subpanel shows prominent spatial frequency of 0.52 µm^−1^ with both α-actinin (green) and stac2 (red). (**G**) Immunoblotting with stac2 antibody shows stac2 expression,~50 kDa band, in HEK293 cells transfected with recombinant stac2, but not in untransfected HEK293 cells. Analysis of freshly dissociated aGPVM confirms basal stac2 expression. Image representative of five independent biological replicates.

**Figure 6—figure supplement 2.**
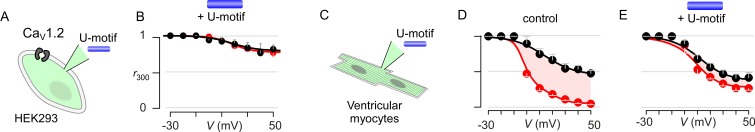
Pipette dialysis of U-motif as a peptide abolishes CDI of both recombinant and native L-type currents in ventricular myocytes. (**A**) Cartoon schematic shows pipette dialysis of U-motif into HEK293 cells expressing recombinant Ca_V_1.2. (**B**) Pipette dialysis of minimal effector stac2 U-motif suppresses Ca^2+^/CaM regulation. Format as in Figure 1—figure supplement 1A. (**C**) Cartoon schematic shows pipette dialysis of U-motif into freshly dissociated adult guinea pig ventricular myocytes that feature endogenous L-type currents. (**D**) Population data show that endogenous L-type Ca^2+^-current in ventricular myocytes elicits strong CDI. Format as in Figure 1—figure supplement 1A. Each symbol, mean ±S.E.M. from six cells. (**E**) In comparison to control conditions, pipette dialysis of minimal effector stac2 U-motif strongly diminishes Ca^2+^/CaM regulation of endogenous Ca^2+^ current in ventricular myocytes. Format as in Figure 1—figure supplement 1A.

### Fhf selectively abrogate CaM signaling to Na_V_1

**Figure 7.**
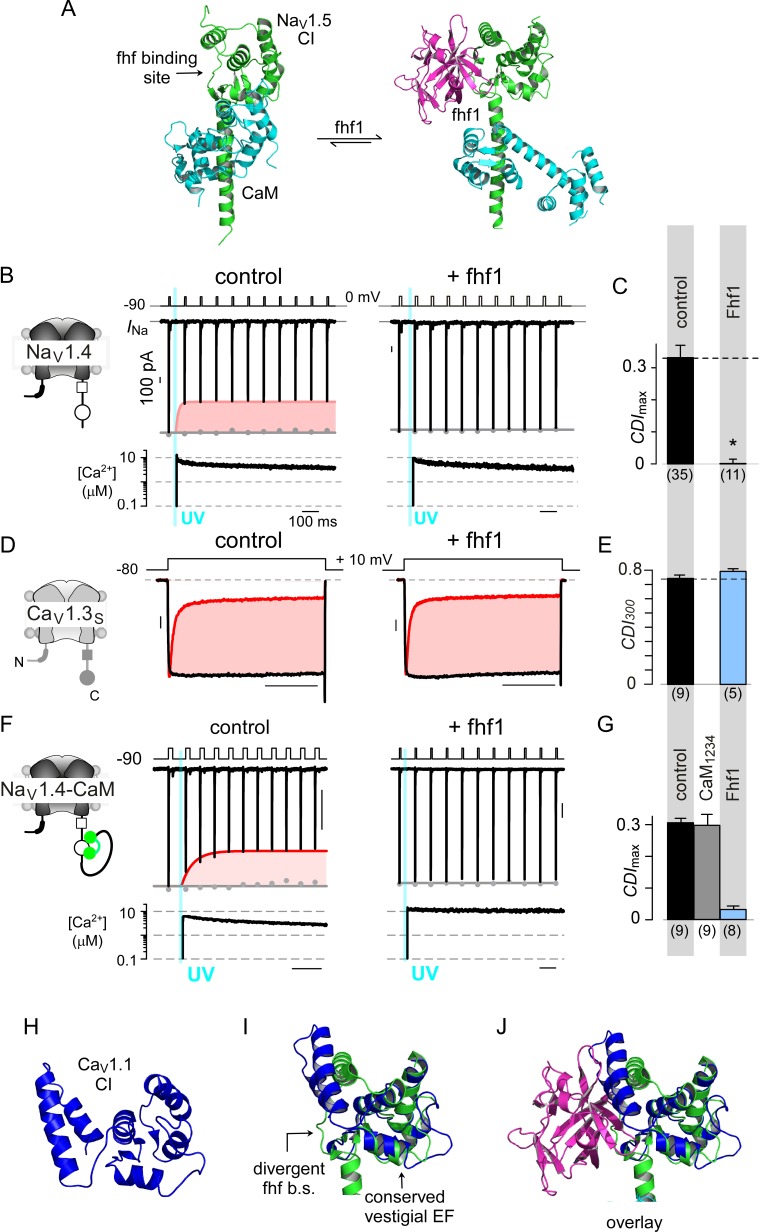
Fhf uses an allosteric mechanism to abrogate Ca^2+^-feedback of Na_V_1.4. (**A**) Structural comparison of Na_V_1.5 CI (green) in the presence of CaM alone (cyan, left) or both CaM (cyan) and fhf1b (purple). Fhf binding changes baseline conformation of CaM on Na_V_1 CI. (**B–C**) Co-expression of fhf1b abolishes CDI in Na_V_1.4 evoked via Ca^2+^ photo-uncaging. Format as in Figure 1M–N. Control data are reproduced from Figure 1M–N for comparison. (**D–E**) In sharp contrast, strong overexpression of fhf1b does not alter CDI of Ca_V_1.3_S_. Format as in Figure 1A–B. Control data reproduced from Figure 1D for comparison. (**F–G**) Fhf1 suppresses CDI of Na_V_1.4 tethered to CaM. Fusion of CaM protects Na_V_1.4 from competitive inhibitors such as CaM_1234_ (**G**). Format as in Figure 1M–N. (**H**) Structure of Ca_V_1.1 upstream CI elements (blue) composed of dual vestigial EF hands and preIQ segments isolated from cryo-EM structure of Ca_V_1.1 (PDBID, 5GJV). This domain is the primary interface for stac interaction in the Ca_V_1 CI. (**I**) Structural overlay of upstream CI elements of Ca_V_1.1 (PDBID, 5GJV) and Na_V_1.5 (PDBID, 4DCK) shows highly conserved dual vestigial EF hand segments while the fhf binding site is structurally divergent. (**J**) The divergence in the fhf binding interface in Ca_V_1.1 in comparison to Na_V_1.5 would introduce a steric clash that prohibits fhf binding to Ca_V_ channels.

Encouraged by the selectivity of stac for Ca_V_1, we sought to identify other regulatory proteins that may tune CaM-signaling to related channel families. However, recognizing such modulators amidst ion channel signalosomes is challenging. Given that stac interacts with Ca_V_1 CI module via the PCI element, we reasoned that other Ca_V_ and Na_V_ interacting proteins that engage a similar interface may suppress CaM-feedback. Intriguingly, recent atomic structures show that fhf interacts with Na_V_1 CI module via the PCI interface (Figure 7A) (Wang et al., 2012). Yet, functionally, fhf isoforms are thought to modulate only voltage-dependent gating properties, with effects on Ca^2+^/CaM-regulation unknown (Goldfarb et al., 2007; Lou et al., 2005; Wang et al., 2012). To test whether fhf alters Na_V_ CDI, we undertook quantitative Ca^2+^ photo-uncaging of the skeletal muscle Na_V_1.4 isoform. We focused here on fhf1b given its modest baseline expression in skeletal muscle and pathological enrichment in critical illness myopathies (Kraner et al., 2012). Figure 7B reproduces baseline levels of CDI for Na_V_1.4 under control conditions. Co-expression of fhf1b abolished CDI (Figure 7B–C; Figure 7—figure supplement 1A), unveiling a novel role of fhf in tuning Ca^2+^-feedback of Na_V_ channels. To assess selectivity, we probed whether fhf alters CDI of Ca_V_1.3. In comparison to control conditions, fhf co-expression spared Ca_V_1.3 CDI (Figure 7D–E; Figure 7—figure supplement 1B) suggesting that fhf may be a selective modulator of Na_V_1.

**Figure 7—figure supplement 1.**
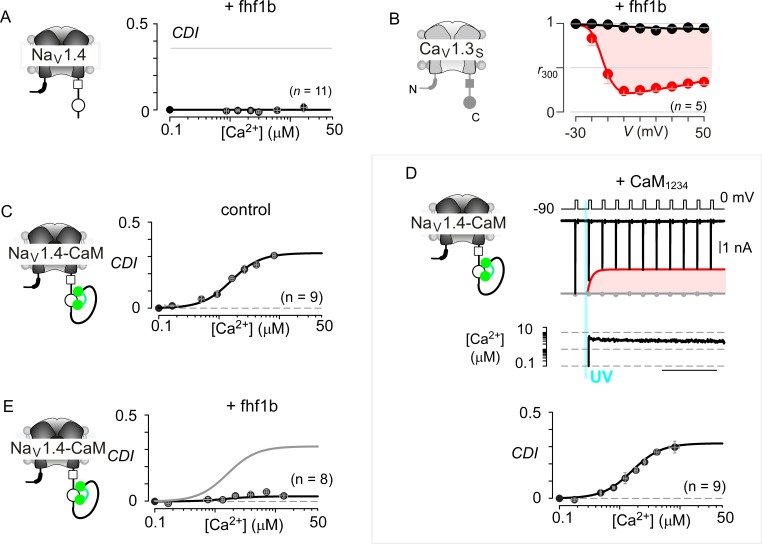
Extended data confirm the selectivity of fhf in modulating Na_V_ versus Ca_V_ channels. (**A**) Population data show that Fhf1 abolishes CDI of Na_V_1.4 channels. Mean data for CDI are plotted versus Ca^2+^-step amplitude. Symbols, mean ±S.E.M. of around four uncaging events compiled from denoted number of cells. (**B**) Fhf does not alter CDI of Ca_V_1.3_S_ demonstrating selectivity of fhf1 in modulating Na_V_ channels. Strong CDI of Ca_V_1.3_S_ persists in the presence of fhf as confirmed by population data of *r*_300_ values. (**C**) Na_V_1.4 fused to CaM (Na_V_1.4-CaM) exhibits strong CDI identical to untethered channels as illustrated by identical fit reproduced from (**A**). (**D**) Fusion of CaM_WT_ protects Na_V_1.4 from CaM_1234_, a competitive inhibitor of CDI. Exemplar currents shows robust CDI of Na_V_1.4-CaM in the presence of CaM_1234_. Format as in Figure 1M and (**A**). (**E**) Fhf1b suppresses CDI of Na_V_1.4-CaM confirming the dominance of fhf over CaM in modulation of Na_V_1.4. Format as in (**A**).

Mechanistically, functional results along with atomic structures of Na_V_1 CI bound to CaM and fhf yield insights on mechanisms for CDI suppression (Gabelli et al., 2014; Wang et al., 2012; Wang et al., 2014). Both fhf and CaM bind concurrently to Na_V_1 CI (Wang et al., 2012; Wang et al., 2014), with fhf binding triggering a conformational rearrangement of CaM (Figure 7A) (Gabelli et al., 2014; Wang et al., 2012). To experimentally validate allostery, we followed our strategy with Ca_V_1.3 and tethered CaM to Na_V_1.4 carboxy-tail. Reassuringly Na_V_1.4-CaM exhibits robust baseline CDI (Figure 7F; Figure 7—figure supplement 1C). Whereas dominant negative CaM_1234_ typically abolishes CDI of Na_V_1.4 (Ben-Johny et al., 2014), Na_V_1.4-CaM exhibits robust CDI despite CaM_1234_, confirming the protective nature of tethered CaM against competitive inhibitors (Figure 7G; Figure 7—figure supplement 1D). Co-expression of fhf1b, however, reduces CDI of Na_V_1.4-CaM (Figure 7F–G; Figure 7—figure supplement 1E). Thus, like stac modulation of Ca_V_1, fhf overrides CaM signaling to Na_V_1.4 despite a tethered CaM, suggesting that fhf acts in allostery.

To garner a structural perspective, we turn to Na_V_1.5 CI/fhf complex (Figure 7A) as the atomistic basis of the stac/Ca_V_1 CI interaction is unknown (Wang et al., 2012; Wang et al., 2014; Wong King Yuen et al., 2017). Whereas the dual-vestigial EF hand segments of Na_V_1.5 and Ca_V_1.1 are similar (Figure 7H–I), the fhf binding interface of Na_V_1.5, including the preIQ loop diverges from corresponding segments of Ca_V_1.1 and introduces a steric clash (Figure 7I–J) (Wang et al., 2012; Wu et al., 2016). Thus, by leveraging structurally distinct loci on the CI module, fhf selectively diminish CaM signaling to Na_V_ channels. These findings point to a class of auxiliary proteins that selectively adjust Ca^2+^-dependent feedback to individual ion channel targets.

### Engineering synthetic modulation of Ca_V_ channels

**Figure 8.**
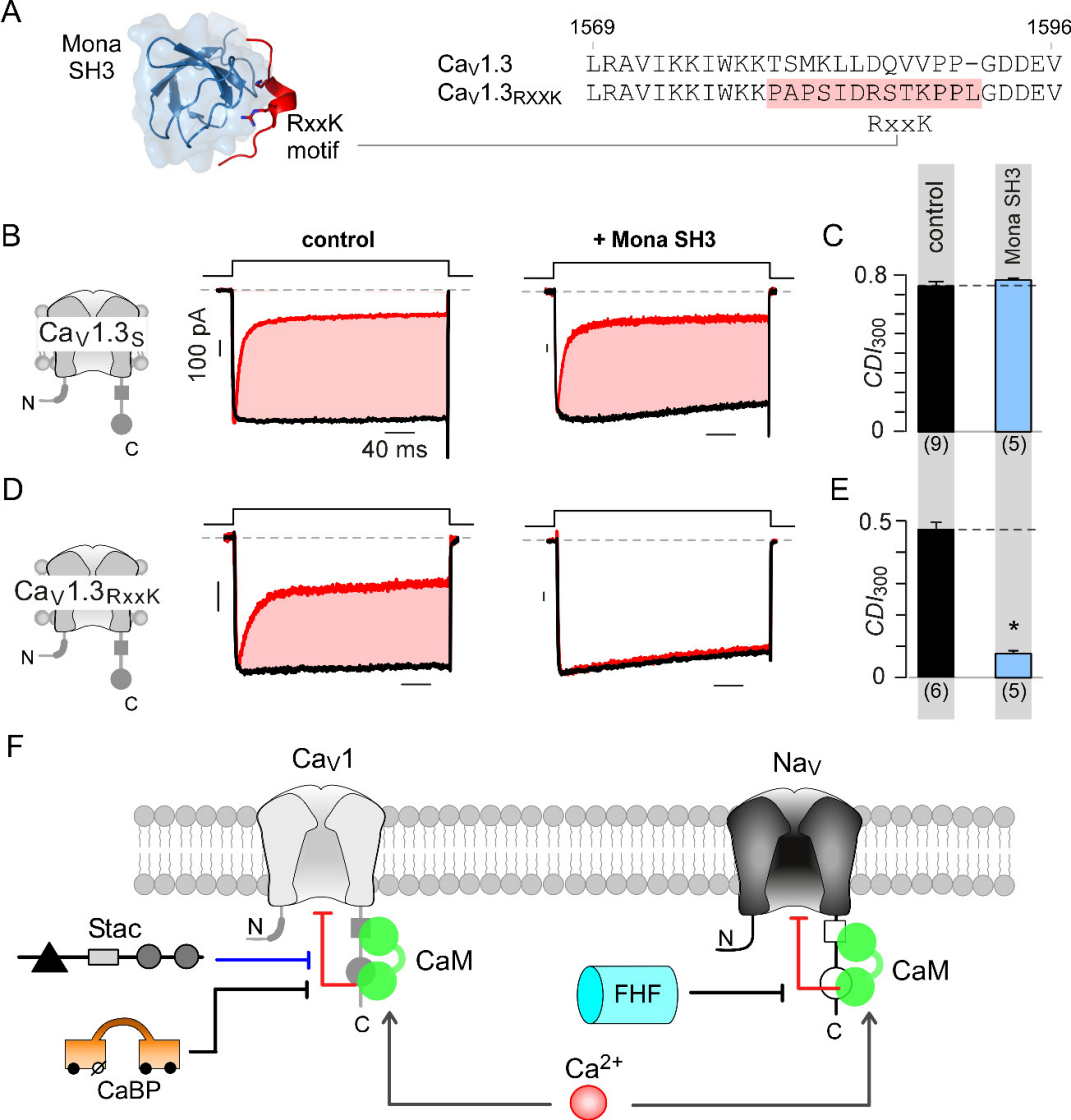
Engineering synthetic modulation of Ca_V_1 channels. (**A**) Left, schematic shows the atomic structure of Mona SH3 domain in complex with RxxK motif. Right, sequence alignment outlines strategy for insertion of RxxK motif into Ca_V_1.3, yielding Ca_V_1.3_RxxK_ to confer synthetic suppression of Ca_V_1.3 CDI by Mona SH3. (**B–C**) Ca_V_1.3_S_ expressed with and without Mona SH3 shows full CDI, confirming that wildtype Ca_V_1.3 CDI is insensitive to Mona SH3. Format as in Figure 1A–B. Control data are reproduced from Figure 1D for comparison. (**D–E**) Mona SH3 strongly diminishes CDI of Ca_V_1.3_RxxK_. Format as in Figure 1A–B. (**F**) Cartoon summarizes selective modulation of Ca^2+^/CaM signaling to Ca_V_1, and Na_V_1 channels with CaM, stac, and fhf.

As both stac and fhf tune Ca^2+^-feedback to individual Ca_V_ and Na_V_ targets by interacting with respective PCI segments, this mechanism furnishes a strategy to engineer synthetic channel modulators. We reasoned that introducing a short interaction motif into the PCI locus may permit inhibition of Ca_V_1 Ca^2+^-feedback by a novel protein. We chose the well-characterized RxxK motif from SLP-76 for its small size and high-affinity interaction with SH3 domain of Mona (Harkiolaki et al., 2003) (Figure 8A). Co-expression of Mona SH3 with wildtype Ca_V_1.3_S_ demonstrated the persistence of CDI, confirming the suitability of these channels as a ‘blank slate’ to confer synthetic modulation (Figure 8B–C; Figure 8—figure supplement 1A). We replaced a 12-residue segment in the preIQ domain with the RxxK motif, as highlighted in Figure 8A, yielding Ca_V_1.3_RxxK_ engineered channels. As this locus is situated upstream of the IQ domain, this maneuver spares apoCaM prebinding. Under endogenous levels of CaM, Ca_V_1.3_RxxK_ exhibit robust baseline CDI (Figure 8D–E; Figure 8—figure supplement 1B). Co-expression of Mona SH3 with Ca_V_1.3_RxxK_ markedly diminished CDI (Figure 8D–E; Figure 8—figure supplement 1B) , thus revealing engineered CDI suppression. These findings illustrate the versatility of the CI module as a regulatory hub and highlight the feasibility of developing synthetic modulators to tune Ca^2+^-feedback of ion channels.

**Figure 8—figure supplement 1.**
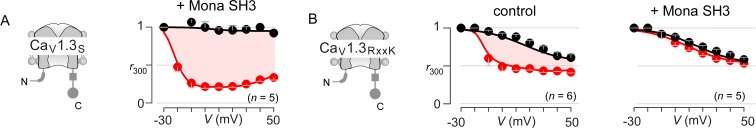
Extended data demonstrate feasibility of engineering synthetic modulators of CaM signaling to Ca_V_1.3. (**A**) Ca_V_1.3_S_ is insensitive to the SH3 domain of Mona/gads protein. Population data of inactivation *r*_300_ values show strong CDI of Ca_V_1.3_S_ in the presence of Mona SH3. Format as in Figure 1—figure supplement 1A. (**B**) In sharp contrast, the introduction of RxxK motif to Ca_V_1.3 upstream CI elements (left subpanel) confers synthetic modulation of CaM signaling by Mona SH3. Middle, population *r*_300_ values show CDI of Ca_V_1.3_RxxK_ under endogenous levels of CaM. Right, co-expression of Mona SH3 abolishes CDI of Ca_V_1.3_RxxK_, illustrating the feasibility of engineering synthetic regulatory proteins that abolish Ca^2+^/CaM signaling to selected targets. Format as in Figure 1—figure supplement 1A.

## Discussion

CaM is a dynamic regulator of Ca_V_1, Ca_V_2, and Na_V_1, affording millisecond-precision Ca^2+^-feedback of channel activity. Our findings suggest that distinct auxiliary regulatory proteins tune CaM signaling to individual targets selectively. Stac prevents CaM signaling to Ca_V_1, while fhf reduces signaling to Na_V_1 (Figure 8F). Parallel analysis of the two proteins delineates mechanisms and sets the stage for in-depth physiological analysis.

### Relationship to prior studies of stac-Ca_V_ modulation

Stac regulation of Ca_V_1 modifies multiple aspects of Ca_V_1 function. For Ca_V_1.1, stac3 enhances plasmalemmal trafficking (Linsley et al., 2017b; Niu et al., 2018; Polster et al., 2015; Wong King Yuen et al., 2017; Wu et al., 2018), and promotes conformational coupling to RyR (Linsley et al., 2017a; Polster et al., 2016). For Ca_V_1.2, however, stac1-3 isoforms slow inactivation (Campiglio et al., 2018; Polster et al., 2015; Wong King Yuen et al., 2017). Our work generalizes the latter effect to the Ca_V_1 family and further identifies a change in baseline channel openings (*P*_O_).

A few mechanistic nuances merit attention. First, stac binds to multiple Ca_V_1 segments including (1) the II-III linker (Polster et al., 2018; Wong King Yuen et al., 2017), (2) the III-IV linker (Figure 2D), and (3) the carboxy-tail (Figure 2D) (Campiglio et al., 2018; Niu et al., 2018). Previous studies have shown that stac interaction with the II-III linker is important for Ca_V_1 trafficking in skeletal muscle (Polster et al., 2018; Wong King Yuen et al., 2017). Chimeric analysis here suggests that stac interaction with the carboxy-tail is critical for tuning CDI. Prior analysis of Ca_V_1.2 triadic localization in myotubes suggested that the channel IQ domain may be important for stac binding (Campiglio et al., 2018). However, FRET 2-hybrid assay indicates that stac interaction with the IQ is around tenfold weaker than with the PCI segment. Second, prior work also suggested that stac-mediated reduction in CDI results from competitive displacement of CaM by stac (Campiglio et al., 2018). Functional experiments using Ca_V_1 tethered to CaM, however, suggest that stac does not compete with CaM. Consistent with this scheme, FRET 2-hybrid analysis shows that CaM binding with the CI module is intact even in the presence of stac. Third, key domains within stac relevant for Ca_V_ modulation remain controversial. Previous studies have identified the dual SH3 and C1 domains to be critical for stac effect on trafficking and coupling to RyR (Campiglio and Flucher, 2017; Linsley et al., 2017a; Linsley et al., 2017b; Polster et al., 2016), while the C1 has been proposed to be critical for modifying Ca_V_1 CDI (Campiglio et al., 2018; Wong King Yuen et al., 2017). Our findings instead suggest that the U-domain in the stac2 linker region is sufficient to fully recapitulate reduction in Ca_V_1 CDI. Notably, prior analysis of the C1 domain also included this linker (Wong King Yuen et al., 2017). Given these experimental findings, a simple possibility is that distinct subdomains within stac interact with disparate channel segments to support multifunctionality of stac. While the U-domain modifies channel inactivation, other subdomains may support plasmalemmal trafficking and conformational coupling.

### Defining a general class of auxiliary modulators of CaM signaling

Although functionally divergent, Ca_V_1, Ca_V_2, and Na_V_1 feature a modular CI element with a common CaM interaction fingerprint and subsequently, shared mechanistic basis for Ca^2+^-regulation. For all three families, apoCaM prebinds the CI module while Ca^2+^/CaM interaction switches channels between discrete high and low *P*_O_ gating modes (Ben-Johny et al., 2015). How do allosteric regulators override CaM-signaling? First, stac and fhf use unique interfaces on the channel CI to selectively tune Ca^2+^-feedback. Second, stac locks Ca_V_1 into a high *P*_O_ gating mode irrespective of whether apoCaM or Ca^2+^/CaM is bound, effectively disengaging the pore from CaM-conformational changes. For Na_V_1, despite fhf binding, CaM undergoes a profound Ca^2+^-dependent rearrangement (Wang et al., 2012; Wang et al., 2014) suggesting that fhf does not prevent Ca^2+^ binding to CaM or Ca^2+^/CaM interaction with effector interfaces. Instead, like stac and Ca_V_1, fhf may override CaM-dependent changes to Na_V_, akin to a clutch disengaging power transmission in mechanical systems. As fhf elicits a change in apoCaM conformation (Figure 7A) (Gabelli et al., 2014; Wang et al., 2012), baseline gating of Na_V_ may also be altered (Goldfarb et al., 2007; Lou et al., 2005). This parallelism between stac and fhf hints at a shared mechanism.

Ca^2+^-binding proteins (CaBPs) (Haeseleer et al., 2000) also suppress CaM signaling to Ca_V_1 (Lee et al., 2002; Yang et al., 2006). Mechanistically, CaBPs exploit a mixed allosteric scheme – at low concentrations, they engage distinct interfaces from CaM but at higher concentrations displace CaM (Findeisen and Minor, 2010; Oz et al., 2013; Yang et al., 2014). The existence of other regulatory proteins that curtail Ca^2+^-feedback points to a general class of auxiliary regulators of CaM-signaling to targets beyond Na_V_1 and Ca_V_1. Identifying such molecular players is critical to understand how CaM signaling is orchestrated.

### Biological implications of stac modulation of Ca_V_1

Stac1/2 isoforms are widely expressed in multiple brain regions, including both the hippocampus and the midbrain (Nelson et al., 2013; Suzuki et al., 1996). Our experiments hint at low basal stac2 expression in guinea pig ventricular cardiac myocytes, although previous studies have failed to detect stac2 in murine heart (Nelson et al., 2013). Further quantitative analysis will help establish ambient stac levels including species-specific differences and potential modulatory effects on cardiac function. Interestingly, endogenous Ca_V_1 in both hippocampal and midbrain neurons (Bazzazi et al., 2013; Oliveria et al., 2012) as well as ventricular cardiac myocytes exhibit CDI. As all stac variants shunted CDI of Ca_V_1 in HEK293, it is possible that stac function may be tightly regulated in native settings. One possibility is that stac abundance may be tuned developmentally (Suzuki et al., 1996), pathologically, or via interacting proteins (Satoh et al., 2006). For instance, the transcription factor, NFAT binds to an upstream promoter region of stac2 gene to upregulate stac2 expression in osteoclasts as well as during hypoxic conditions in neural stem cells (Jeong et al., 2018; Moreno et al., 2015). Physiologically, as Ca_V_1 CDI is a potent homeostatic mechanism that prevents pathological Ca^2+^-overload (Dunlap, 2007), a low concentration regime of stac may be advantageous. By modulating a subpopulation of Ca_V_1, stac may circumvent homeostatic requirements to amplify local Ca^2+^-signals via sustained Ca^2+^ influx. The C1 and SH3 domains may serve as scaffolds to localize stac to specific signaling complexes (Campiglio and Flucher, 2017; Cohen et al., 1995; Colon-Gonzalez and Kazanietz, 2006). It is also possible that phosphorylation of stac may dynamically tune its function (Huttlin et al., 2010). Resolving these complexities may unveil mechanisms that tune Ca_V_ function spatially and temporally.

In cardiac myocytes, CDI of Ca_V_1 is a key factor for action potential duration (Limpitikul et al., 2014; Mahajan et al., 2008). Experimentally, this importance is inferred from prolongation of action potentials upon expression of mutant CaM_1234_ (Alseikhan et al., 2002). Yet, constitutive CaM expression may yield nonspecific effects (Hall et al., 2013; Wang et al., 2007) that obscure the net contribution of Ca_V_1 CDI (Zhang et al., 2015). Acute elevation of the U-domain bypasses these ambiguities and confirms a key role for Ca_V_1 CDI for cardiac action potentials. Pathophysiologically, differential expression of stac2 has been reported in right ventricular heart failure, hinting at a potential role in calcium remodeling during heart failure (di Salvo et al., 2015).

Post-transcriptional modification of Ca_V_1.3 generates an assortment of variants with modified carboxy-termini (Bock et al., 2011; Huang et al., 2012). The apoCaM affinities of these variants are such that CaM fluctuations may redistribute channels between populations lacking or endowed with apoCaM (Bazzazi et al., 2013), evoking concomitant changes in maximal *P*_O_ and CDI of Ca_V_1.3 (Adams et al., 2014). Stac uniformly locks these variants into a high *P*_O_ configuration incapable of CDI, thereby supporting reliable and persistent Ca^2+^-influx in spite of CaM. Notably, functional effects of Ca_V_1.3 alternative splicing have been shown to be cell-type specific suggesting that auxiliary regulators may tune channel properties (Scharinger et al., 2015). Fitting with these regulatory possibilities, disruption of stac modulation of Ca_V_1.3 in Drosophila alters circadian rhythm (Hsu et al., 2018).

### Biological implications of fhf modulation of Na_V_1

Unlike canonical fibroblast growth factors, fhf lack a secretory signal sequence (Smallwood et al., 1996) and serve as intracellular proteins (Schoorlemmer and Goldfarb, 2001). Four distinct fhf isoforms have been identified with tissue-specific expression in neurons, cardiomyocytes, and skeletal muscle (Goldfarb, 2005; Kraner et al., 2012; Smallwood et al., 1996). Functionally, fhf isoforms promote Na_V_1 trafficking and fast inactivation (Pablo and Pitt, 2016). More specifically, fhf adjust steady-state voltage-dependence of inactivation (Lou et al., 2005), elicit a kinetically distinct long-term inactivation (Dover et al., 2010), and modify resurgent current (Yan et al., 2014). Our present findings suggest that fhf1 also tunes CDI of Na_V_1. Physiologically, Na_V_ CDI may be prominent during repetitive activity, as excess Ca^2+^ accumulation may inhibit Na currents. Thus, suppression of Na_V_1 CDI by fhf may enhance repetitive firing. Interestingly, loss of fhf1 and/or fhf4 result in diminished firing properties of cerebellar Purkinje neurons (Bosch et al., 2015; Goldfarb et al., 2007), while loss of fhf2 reduces cardiac conduction (Park et al., 2016; Wang et al., 2011a). It is possible that loss of fhf may enhance net CDI thus contributing to diminished excitability in these cells. As mutations in fhf1 are associated with epileptic encephalopathy (For CENetDDD Study group‡* et al., 2016) and cardiac conduction disorders (Hennessey et al., 2013) while mutations in fhf4 are linked to spinocerebellar ataxia (Brusse et al., 2006), resolving the dynamic interplay between CaM and fhf in tuning Na_V_1 may be critical for understanding pathogenic mechanisms.

### New strategy for synthetic ion channel modulation

Finally, our results highlight the possibility of engineering synthetic regulation to tune CaM signaling. While Ca_V_1.3 is insensitive to Mona SH3, insertion of an RxxK motif (Harkiolaki et al., 2003) into the carboxy-tail preIQ segment allows latent modulation by Mona SH3. Given the structural similarity of the CI modules of Ca_V_1, Ca_V_2, and Na_V_1, and sequence variability within the preIQ domain, emerging protein engineering methods may be used to screen for synthetic modulators of related ion channel families. As the ligand specificity of SH3 domains can be custom-engineered (Nguyen et al., 2000) and subcellular localization tuned via targeting motifs (Komatsu et al., 2010), a custom library of synthetic regulators may be developed to combinatorially modify kinetic properties of Ca_V_1, Ca_V_2, or Na_V_1 channels with spatiotemporal specificity. Generalizing this approach may lead to the development of new tools to manipulate Ca^2+^ signaling.

In all, our findings unravel the elegant interplay between a novel class of allosteric regulators and CaM in orchestrating the activity of Ca_V_ and Na_V_ channels.

## Materials and methods

**Key resources table:** 

Reagent type (species) or resource	Designation	Source or reference	Identifiers	Additional information
Gene (rat)	β_2A_	PMID: 1370480	GenBank: M80545	
Gene (rat)	α_2_δ	PMID: 8107966	NCBI: NM_012919	
Gene (*Oryctolagus cuniculus*)	Ca_V_1.2	PMID: 1718988	NCBI: NM_001136522	
Gene (rat)	Ca_V_1.3_S_	PMID: 20139964	GenBank: AF370009.1	
Gene (rat)	Ca_V_1.3_MQDY_	PMID: 24120865, 22284185		
Gene (human)	Ca_V_1.4_43*_	PMID: 22069316		Laboratory of Dr. Soong Tuck Wah (National University of Singapore)
Gene (human)	Ca_V_2.1 splice variant 37a(EFa) with 43^+^/44^-^/47^-^	PMID: 12451115		
Gene (human)	Ca_V_2.2	PMID: 1321501, 10233069	GenBank: M94172.1	
Gene (rat)	Ca_V_2.3	PMID: 8388125, 18400181	NCBI: NM_019294.2	
Gene (rat)	Na_V_1.4	PMID: 2175278		
Gene (human)	stac1	Origene	NCBI: NP_003140.1	
Gene (mouse)	stac2	Origene	NCBI: NP_666140.1	
Gene (human)	stac3	Origene	NCBI: NP_659501.1	
Gene (human)	fhf	PMID: 8790420		Laboratory of Dr. Jeremy Nathans (Johns Hopkins University).
Gene (human)	Mona SH3	PMID: 12773374		Synthesized by Genscript based on sequence in publication
Peptide (mouse)	U-peptide	this paper		Peptide sequence KVDPVYETLRYGTSLALM NRSS synthesized by Genscript
Competent cells (*E. coli*)	DH5α	Invitrogen: 18265017		
Cell line (human)	HEK293	other	RRID: CVCL_0045	
Biological sample (guinea pig)	aGPVM	PMID: 24076394		Generated from Hartley strain guinea pigs
Antibody	anti-stac1	Abcam: ab181157		1:100
Antibody	anti-stac2	Abcam: ab156080		IHC – 1:100 WB – 1:250
Antibody	anti-α-actinin	Sigma Aldrich: A7811	RRID: AB_476766	1:300
Antibody	goat anti- rabbit IgG Alexa Fluor 594	Abcam: ab150080	RRID: AB_2650602	1:1000
Antibody	goat anti-mouse IgG1 Alexa Fluor 488	Thermo Fischer: A21121	RRID: AB_2535764	1:1000
Antibody	Goat Anti-Rabbit IgG (H + L)	Jackson ImmunoResearch: 111-035-144	RRID: AB_2307391	1:10,000
Recombinant DNA reagent	Ca_V_1.3_L_	PMID: 20139964		Engineered from Ca_V_1.3_S_ and human long distal carboxyl tail (NCBI: NM_000718)
Recombinant DNA reagent	Ca_V_2.3/1.3 CI	PMID: 24441587		
Recombinant DNA reagent	Ca_V_1.3- CaM_WT_	PMID: 24441587		
Recombinant DNA reagent	Ca_V_1.2- CaM_WT_	PMID: 15087548		
Recombinant DNA reagent	Na_V_1.4-CaM	this paper		Engineered by fusing CaM_WT_ carboxy-tail of Na_V_1.4
Recombinant DNA reagent	Ca_V_1.3_RxxK_	this paper		Engineered from Ca_V_1.3_S_
Recombinant DNA reagent	CFP-stac3	this paper		stac3 was cloned into CFP vector with *NotI* and *XbaI*
Recombinant DNA reagent	YFP-Ca_V_1.3 CI	PMID: 23591884		
Recombinant DNA reagent	YFP-Ca_V_1.3 PCI	PMID: 23591884		
Recombinant DNA reagent	YFP-Ca_V_1.3 IQ	PMID: 23591884		
Recombinant DNA reagent	Ven-C1	this paper		stac2 C1 was cloned into Venus vector (PMID: 26997269) with *NotI* and *XbaI*
Recombinant DNA reagent	Ven-linker region	this paper		stac2 linker region was cloned into Venus vector (PMID: 26997269) with *NotI* and *XbaI*
Recombinant DNA reagent	Ven-SH3-SH3	this paper		stac2 SH3-SH3 was cloned into Venus vector (PMID: 26997269) with *NotI* and *XbaI*
Recombinant DNA reagent	Ven-U-motif	this paper		stac2 U-motif was cloned into Venus vector (PMID: 26997269) with *NotI* and *XbaI*
Recombinant DNA reagent	stac2 (KVD/AAA)	this paper		Quickchange PCR with stac2
Recombinant DNA reagent	stac2 (PVY/AAA)	this paper		Quickchange PCR with stac2
Recombinant DNA reagent	stac2 (ETL/AAA)	this paper		Quickchange PCR with stac2
Recombinant DNA reagent	stac2 (RYG/AAA)	this paper		Quickchange PCR with stac2
Recombinant DNA reagent	stac2 (TSL/AAA)	this paper		Quickchange PCR with stac2
Recombinant DNA reagent	stac2 (NRS/AAA)	this paper		Quickchange PCR with stac2
Recombinant DNA reagent	stac2 (S/A)	this paper		Quickchange PCR with stac2
Sequence-based reagent	Ven-C1 forward primer	this paper		cttctcgcggccgc tatgaccgaa atga gcgagaa
Sequence-based reagent	Ven-C1 reverse primer	this paper		tcagaattctagattat tgctggt gggagatctc
Sequence-based reagent	Ven-linker region forward primer	this paper		cttctcgcggccgcta catctttt cgacgcaact
Sequence-based reagent	Ven-linker region reverse primer	this paper		tcagaattctagatta gtacatg ggccccacg
Sequence-based reagent	Ven-SH3-SH3 forward primer	this paper		cttctcgcggccgc ttcctacgt cgccctct
Sequence-based reagent	Ven-SH3-SH3 reverse primer	this paper		tcagaattctagattat cagatctct gccaaggag
Sequence-based reagent	Ven-U-motif forward primer	this paper		cttctcgcggccgctaagg tggac ccagtttatga
Sequence-based reagent	Ven-U-motif reverse primer	this paper		tcagaattctagattag ctggaa cggttcatcag
Sequence-based reagent	stac2 (KVD/AAA) sense	this paper		ctactgggaccagcgg ggcggcgg ccccagt ttatgagacgc
Sequence-based reagent	stac2 (KVD/AAA) antisense	this paper		gcgtctcataaactggg gccgccgc cccgctgg tcccagtag
Sequence-based reagent	stac2 (PVY/AAA) sense	this paper		ccagcgggaaggtggac gcagc tgctgagacgct gcgctatg
Sequence-based reagent	stac2 (PVY/AAA) antisense	this paper		catagcgcagcgtctc agcagct gcgtccacc ttcccgctgg
Sequence-based reagent	stac2 (ETL/AAA) sense	this paper		ggtggacccagttt atgcggcgg cgcgct atggcacctcc
Sequence-based reagent	stac2 (ETL/AAA) antisense	this paper		ggaggtgccatagcgc gccgcc gcataaact gggtccacc
Sequence-based reagent	stac2 (RYG/AAA) sense	this paper		cccagtttatgagacgc tggccgc tgccacctcc ctggcactgatg
Sequence-based reagent	stac2 (RYG/AAA) antisense	this paper		catcagtgccaggg aggtggca gcggccagc gtctcataaactggg
Sequence-based reagent	stac2 (TSL/AAA) sense	this paper		acgctgcgctatgg cgccgccgc ggcactga tgaaccg
Sequence-based reagent	stac2 (TSL/AAA) antisense	this paper		cggttcatcagtgc cgcggcggc gccatag cgcagcgt
Sequence-based reagent	stac2 (NRS/AAA) sense	this paper		gatgtgctgctga agctggcagcg gccatc agtgccagggaggtg
Sequence-based reagent	stac2 (NRS/AAA) antisense	this paper		cacctccctggc actgatggccgc tgcc agcttcagcagcacatc
Sequence-based reagent	stac2 (S/A) sense	this paper		cactgatgaacc gttccgccttc agcag cacatctg
Sequence-based reagent	stac2 (S/A) antisense	this paper		cagatgtgctgctga aggcgg aacggttca tcagtg
Software, algorithm	PyMOL	http://www.pymol.org/	RRID: SCR_000305	

### Molecular biology and peptide synthesis

Ca_V_1.2, Ca_V_1.3, Ca_V_1.4_43*_, Ca_V_2.1, Ca_V_2.2, Ca_V_2.3, and Na_V_1.4 variants were unmodified from previously published constructs: Ca_V_1.2 (NM001136522) (Wei et al., 1991), Ca_V_1.2-CaM_WT_ (Mori et al., 2004), Ca_V_1.3_S_ (AF370009.1), Ca_V_1.3_L_ engineered from Ca_V_1.3_S_ and human long distal carboxyl tail (NM000718) (Liu et al., 2010), RNA-edited variant Ca_V_1.3_MQDY_ (Bazzazi et al., 2013; Huang et al., 2012), Ca_V_1.4_43*_ was gifted from Dr. Soong Tuck Wah (National University of Singapore), Ca_V_2.1 splice variant 37a(EFa) with 43^+^/44^-^/47^−^ (Soong et al., 2002) was gifted from Dr. Terry Snutch (University of British Columbia), Ca_V_2.2 (Jones et al., 1999), Ca_V_2.3 (Mori et al., 2008), Na_V_1.4 (Trimmer et al., 1990). Stac variants were purchased from Origene: human stac1 mRNA transcript 1 (NP003140.1), mouse stac2 (NP666140.1), and human stac3 isoform 2 (NP659501.1). U-peptide was synthesized by Genscript (KVDPVYETLRYGTSLALMNRSS). Fhf variants were gifted from Dr. Gordon Tomaselli and Dr. Jeremy Nathans (Johns Hopkins University).

### Cell culture and transfection of HEK293 cells

For whole-cell electrophysiology, single-channel electrophysiology, and immunohistochemistry, HEK293 cells (ATCC; mycoplasma tested negative) were cultured on glass coverslips in 10 cm dishes and transfected by a calcium phosphate method (Peterson et al., 1999) with the following amounts of DNA: 3 µg of SV40 T antigen to enhance expression, 2–8 µg of α_1_-subunit of Ca^2+^ or Na^+^ channel depending on expression, 8 µg from rat β_2A_ (Perez-Reyes et al., 1992) (M80545), 8 µg from rat α_2_δ (Tomlinson et al., 1993) (NM012919.2), and 8 µg of the stac1, stac2, or stac3 variants indicated.

For FRET two-hybrid experiments, cells were cultured on glass-bottom dishes and transfected with a standard polyethylenimine protocol (Lambert et al., 1996). Epifluorescence measurements were recorded 1–2 days after transfection.

### Adult guinea pig ventricular myocyte isolation

Adult guinea pig ventricular myocytes (aGPVMs) were isolated from whole hearts of Hartley strain guinea pigs 3–4 weeks old (250–350 g). Guinea pigs were anesthetized via intraperitoneal injection with pentobarbital (35 mg/kg). Hearts were then excised, and single ventricular myocytes were isolated following a previously published protocol (Joshi-Mukherjee et al., 2013). Cells were plated on glass coverslips that were laminin (20 µg/mL) coated overnight at 4°C.

Immunohistochemistry aGPVMs plated on glass coverslips were first washed three times with cold PBS and then fixed in 3.7% paraformaldehyde (15710, Electron Microscopy Sciences) in PBS for 15 min. After washing three times with PBS, cells were permeabilized in cold 0.5% Triton X-100 in tris buffered saline (TBS) for 20 min and then blocked with 10% goat serum in PBS for 1 hr at room temperature. Cells were incubated overnight at 4°C in primary antibodies diluted in antibody diluent solution (IW-1000, IHC World): monoclonal anti-α-actinin (sarcomeric) antibody produced in mouse (1:300, A7811), anti-STAC (stac1) antibody [EPR12805]-N-terminal (1:100, ab181157) or anti-STAC2 (stac2) antibody-N-terminal (1:100, ab156080) produced in rabbit. Next day, cells were rinsed three times with 0.05% TWEEN20 (Sigma P9416) in TBS (TBS-T) for 5 min each. In the dark, cells were incubated with secondary antibodies (1:1000): goat anti-mouse IgG1 Alexa Fluor 488 (1:1000, A21121), goat anti-rabbit IgG Alexa Fluor 594 (1:1000), and DAPI (1:10000) diluted in antibody solution for 45 min at room temperature and then washed three times with TBS-T for 5 min each. Stained cells were mounted with prolong gold mounting media (Invitrogen) on a microscope slide (Fischer Scientific).

Transfected HEK293 were immunostained following a similar protocol to that of aGPVM, but were not labelled with sarcomeric primary antibody and its respective secondary antibody.

Western blot aGPVMs and HEK293 cells were washed twice with PBS buffer. Cells were harvested with 1 mL 1x RIPA buffer (20–188, Sigma Aldrich) containing half a tablet of complete mini-EDTA-free protease inhibitor (11836170001, Sigma Aldrich) and incubated at 4°C for 30 min. Samples were centrifuged at 15,000 RPM for 15 min, and the pellet was discarded. Then, 2–5 µg of proteins in the supernatant were heated at 37°C for 30 min with 2x Laemmli sample buffer (S3401, Sigma Aldrich). Samples were loaded into 4–12% gradient gel (NP0335BOX, Invitrogen) with PageRuler plus prestained protein ladder (26619, Invitrogen) and run at 100 V for 2 hr at room temperature in running buffer: 1x NuPAGE MOPS SDS running buffer: 50 mM MOPS, 50 mM Tris base, 0.1% SDS, 1 mM EDTA, pH to 7.7. Proteins were transferred on ice from the gel to nitrocellulose membrane (10600003, GE Healthcare Life science) for 75 min at 10 V in transfer buffer: 24 mM Tris base, 192 mM glycine, 20% v/v methanol. Membrane was blocked with 5% (w/v) Blotting-Grade-Blocker (1706404, Bio-Rad) in 1x TRIS-buffered saline for 1 hr at 4°C. Primary antibody for stac2 (1:250) was added to the blocking buffer with 0.1% (v/v) Tween 20 (1706404, Bio-Rad) and incubated overnight at 4°C. Next day, the membrane was washed three times for 5 min each with TBS with 0.1% (v/v) Tween 20 (TBS-T). The secondary antibody (111-035-144, Jackson ImmunoResearch; 1:10,000) was added to the blocking buffer with 0.1% (v/v) Tween 20 and incubated for 1 hr. The membrane was washed again three times for 5 min each with TBS-T. Finally, western blots were developed with SuperSignal West Pico Chemiluminescent Substrate (34580, ThermoFischer) and images were collected on an Alpha InnoTech FluorChem HD2 imaging system.

### Confocal optical imaging

Images of immunostained tissue slices and cells were captured with either an Olympus Fluorview FV300 confocal laser scanning microscope or an LSM780 (Carl Zeiss, Oberkochen, Germany) confocal microscope. For the FV300, we used Fluoview software (Olympus) with a PlanApo 403 or 603 oil objective (NA 1.40, PLAPO60XO3; Olympus). Argon laser (488 nm) was used to excite Alexa Fluor 488 (green), and Helium Neon (HeNe) Green Laser was used to excite Alexa Fluor 594 (red). Olympus optical filters used were 442/515 nm excitation splitter (FV-FCV), 570 nm emission splitter (FV-570CH), BA510 IF and BA530RIF for green emission channel, and 605 BP ﬁlter for red channel. Images were processed in ImageJ. Similar settings were used for the LSM780 setup.

### Whole-cell electrophysiology

Whole-cell voltage-clamp recordings for HEK293 were collected at room temperature 1–2 days after transfection with Axopatch 200A (Axon Instruments). Glass pipettes (BF150-86-10, Sutter Instruments) were pulled with a horizontal puller (P-97; Sutter Instruments Company) and fire polished (Microforge, Narishige, Tokyo, Japan) to have 1–3 MΩ resistance. Recordings were low-pass filtered at 2 kHz and sampled at 10 kHz with P/8 leak subtraction and 70% series resistance and capacitance compensation. For recordings of Ca_V_1.2 (Figure 1A–B, Figure 5I–K, Figure 6B), Ca_V_1.3_S_ (Figure 1C–D, Figure 3—figure supplement 1A–B and and G, Figure 7D–E, and Figure 8B–C), Ca_V_1.4_43*_ (Figure 1E–F), Ca_V_2.2 (Figure 1I–J), Ca_V_2.3/1.3 CI chimera (Figure 2E–F), Ca_V_1.3-CaM (Figure 3A–B, Figure 3—figure supplement 1C–F, and Figure 5G–H), Ca_V_1.2-CaM (Figure 2C–D, Figure 5A–F) and Ca_V_1.3_RxxK_ (Figure 8D–E) exogenously expressed in HEK293 cells, the internal solution contained (in mM): CsMeSO_3_, 114; CsCl_2_, 5; MgCl_2_, 1; MgATP, 4; HEPES, 10; BAPTA, 10; adjusted to 295 mOsm with CsMeSO_3_ and pH 7.4 with CsOH. The external solution contained (in mM): TEA-MeSO_3_, 140; HEPES, 10; CaCl_2_, or BaCl_2_ 40; adjusted to 300 mOsm with TEA-MeSO_3_ and pH 7.4 with TEA-OH. For recordings of Ca_V_2.1 (Figure 1G–H) and Ca_V_2.3 (Figure 1K–L), the internal solution contained (in mM): CsMeSO_3_, 135; CsCl_2_, 5; MgCl_2_, 1; MgATP, 4; HEPES, 10; EGTA, 1; adjusted to 295 mOsm with CsMeSO_3_ and pH 7.4 with CsOH. The external solution contained (in mM): TEA-MeSO_3_, 140; HEPES, 10; CaCl_2_, or BaCl_2_ 5; adjusted to 300 mOsm with TEA-MeSO_3_ and pH 7.4 with TEA-OH. At a holding potential of −80 mV, we used a family of test pulses from −30 mV to +50 mV with repetition intervals of 20 s. Custom MATLAB (Mathworks) software (https://github.com/manubenjohny/WCDTY; copy archived at https://github.com/elifesciences-publications/WCDTY) was used to determine peak current and fraction of peak current remaining after either 300 ms (*r*_300_) or 800 ms (*r*_800_) of depolarization.

We incubated aGPVMs for 20–48 hr after isolation in 5 µM ryanodine for 5–10 min before we collected whole-cell recordings. The internal recording solution contained (in mM) CsMeSO_3_, 114; CsCl_2_, 5; MgCl_2_, 1; MgATP, 4; HEPES, 10; BAPTA, 10; ryanodine, 0.005 adjusted to 295 mOsm with CsMeSO_3_ and pH 7.4 with CsOH. Cells were sealed in Tyrodes solution, which contained (in mM): NaCl, 135; KCl, 5.4; CaCl_2_, 1.8; MgCl_2_, 0.33; NaH_2_PO_4_, 0.33; HEPES, 5; glucose, 5 (pH 7.4). For CDI measurements, external solutions containing (in mM): TEA-MeSO_3_, 140; HEPES, 10; CaCl_2_, or BaCl_2_ 40; adjusted to 300 mOsm with TEA-MeSO_3_ and pH 7.4 with TEA-OH were perfused. Welch’s T-test was used to verify statistical significance among the population data.

For CDI recordings, we determined required sample size based on power analysis. Based on historical estimates of normal variation in CDI/CDF measurements, we computed the sample size required such that type I and type II errors are 5% to be 3.5. Thus, we obtained at least four independent measurements for all electrophysiological experiments.

Current-clamp recordings of aGPVMs were performed on the same setup and were filtered at 5 kHz and sampled at 25 kHz. The internal solution contained (in mM): K glutamate, 130; KCl, 9; NaCl, 10; MgCl_2_, 0.5; EGTA, 0.5, MgATP, 4; HEPES, 10; adjusted to pH 7.3 with KOH. The external solution contained (in mM): NaCl, 135; KCl, 5.4; CaCl_2_, 1.8; MgCl_2_, 0.33; NaH_2_PO_4_, 0.33; HEPES, 5; glucose, 5 (pH 7.4). The time from upstroke to 80% repolarization (*APD*_80_) was measured with MATLAB (Mathworks) and used as a metric for comparing physiological output between peptide treated and untreated. For experiments with U-peptide, peptide was dissolved in ddH_2_O to 2 mg/mL and then diluted to 500 μM in the appropriate internal solution.

### Single-channel electrophysiology

Single-channel recordings were performed at room temperature using an on-cell configuration previously established in the laboratory (Tay et al., 2012) with the same setup as used for whole-cell electrophysiology. Glass pipettes were pulled and polished from ultra-thick-walled borosilicate glass (BF200-116-10, Sutter Instruments) and coated with sylgard to have 5–10 MΩ resistance. Recordings were filtered at 2–5 kHz. The pipette solution contained (in mM): TEA-MeSO_3_, 140; HEPES, 10; BaCl_2_ 40; adjusted to 300 mOsm with TEA-MeSO_3_ and pH 7.4 with TEA-OH. The external solution contained (in mM): K glutamate, 132; KCl, 5; NaCl, 5; MgCl_2_, 3; EGTA, 2; HEPES, 10; adjusted to 300 mOsm with glucose and pH 7.4 with KOH. Cell-attached single-channel currents were measured during 200 ms voltage ramps between −80 and +70 mV (portions between −50 and 40 mV displayed and analyzed) as previously described. For each patch, we recorded 80–150 sweeps with a repetition interval of 12 s. Patches were analyzed as follows: (1) The leak for each sweep was fit and subtracted from each trace. (2) The unitary current relation, *i*(*V*), was fit to the open-channel current level using the following equation:

$i(V)=-g\cdot (V-V_{\text{S}})\cdot \exp(-(V-V_{\text{S}})\cdot z\cdot F/(R\cdot T))/(1-\exp(-(V-V_{\text{S}})\cdot z\cdot F/(R\cdot T)))$ where *g* is the single-channel conductance (~0.2 pA/mV), *z* is the apparent valence of permeation (~2.1), *F* is Faraday’s constant, *R* is the gas constant, and *T* is the temperature in degrees Kelvin (assumed room temperature). These parameters were held constant for all patches, except for slight variations in the voltage-shift parameter *V*_s_ ~ 35 mV, as detailed below. (3) All leak-subtracted traces for each patch were averaged (and divided by the number of channels in the patch) to yield an *I–V* relation for that patch. As slight variability in *V*_S_ was observed among patches, we calculated an average *V*_S_ for each construct, *V*_S,AVE_. The data from each patch were then shifted slightly in voltage by an amount Δ*V* = *V*_S,AVE_ – *V*_S_, with Δ*V* typically about ±5 mV. This maneuver allowed all patches for a given construct to share a common open-channel GHK relation. Thus shifted, the *I–V* relations obtained from different patches for each condition/construct were then averaged together. (4) *P*_O_ at each voltage was determined by dividing the average *I* (determined in step three above) into the open-channel GHK relation. Channel number was determined by the maximal number of overlapping opening events upon application of the channel agonist Bay K8644 (5 μM) at the end of each recording. For modal analysis, a dashed line discriminator was chosen to be the average single-trial *P*_O_ = 0.075 such that traces with average single-trial *P*_O_ >0.075 were categorized as high *P*_O_ while the remaining traces were considered to be low *P*_O_.

### Quantitative calcium photo-uncaging

All Ca^2+^-uncaging experiments were conducted on a Nikon TE2000 inverted microscope with a Plan Fluor Apo 40 × oil objective as previously described (Ben-Johny et al., 2014). Briefly, a classic Cairn UV flash photolysis system was used for Ca^2+^-uncaging with brief UV pulses of ~1.5 ms in duration powered by a capacitor bank of up to 4000 μF charged to 200–290V. For concurrent Ca^2+^ imaging, Fluo4FF and Alexa568 dyes were dialyzed via patch pipette and imaged using Argon laser excitation (514 nm). Background fluorescence for each cell was measured prior to pipette dialysis of dyes and subtracted subsequently. A field-stop aperture was used to isolate fluorescence from individual cells. Dual-color fluorescence emission was attained using a 545DCLP dichroic mirror, paired with a 545/40 BP filter for detecting Fluo4FF, and a 580LP filter for detecting Alexa568. Typically, uncaging experiments were conducted after ~2 min of dialysis of internal solution. Welch’s T-test was used to verify statistical significance between the population data.

For all Ca^2+^-uncaging experiments, the internal solution contained (in mM): CsMeSO_3_, 120; CsCl, 5; HEPES (pH 7.4 with CsOH), 10; Fluo-4FF pentapotassium salt (Invitrogen), 0.01; Alexa 568 succinimidyl ester (Invitrogen), 0.0025; Citrate, 1; DM-Nitrophen EDTA (DMN) and CaCl_2_ were adjusted to obtain the desired Ca^2+^ flash. Typically, for flashes in the range 0.5–2 μM, DMN, 1 mM; and CaCl_2_, 0.7 mM. For the 2–8 μM range, DMN, 2 mM; and CaCl_2_, 1.4 mM. For larger Ca^2+^ steps, DMN, 4 mM; and CaCl_2_, 3.2 mM. As DMN can bind Mg^2+^, all experiments were conducted with 0 mM Mg^2+^ internally. For all Na channel experiments, the bath solution contained (in mM): TEA-MeSO_3_, 45; HEPES (pH 7.4), 10; NaCl, 100; at 300 mOsm, adjusted with TEA-MeSO_3_.

### FRET-two-hybrid assay

To collect a range of donor molecule (*D*_free_) concentrations, HEK293 cells were transfected with combinations of DNA ratios. Cells were immersed in 2 mM Ca^2+^ Tyrodes solution, which contained (in mM): NaCl, 138; KCl, 4; CaCl_2_, 2; MgCl_2_, 1; HEPES, 10; glucose, 10. Three-cube FRET fluorescence measurements were performed under resting Ca^2+^ concentrations on an inverted fluorescence microscope. FRET efficiency (*E*_A_ and *E*_D_) was calculated for each cell (Erickson et al., 2001) and a binding curve, either *E*_A_ = [*D*_free_]/(*K*_d,EFF_ + [*D*_free_]) · *E*_A,max_ or *E*_D_ = [*A*_free_]/(*K*_d,EFF_ + [*A*_free_]), was fit to compute the effective dissociation constant (*K*_d,EFF_).

## Additional files

Transparent reporting form

### Data availability

All data generated or analysed during this study are included in the manuscript and supporting files.
